# DNMT1 inhibition by pUG-fold quadruplex RNA

**DOI:** 10.1261/rna.079479.122

**Published:** 2023-03

**Authors:** Linnea I. Jansson-Fritzberg, Camila I. Sousa, Michael J. Smallegan, Jessica J. Song, Anne R. Gooding, Vignesh Kasinath, John L. Rinn, Thomas R. Cech

**Affiliations:** 1 BioFrontiers Institute, University of Colorado Boulder, Boulder, Colorado 80303, USA; 2 Department of Biochemistry, University of Colorado Boulder, Boulder, Colorado 80303, USA; 3 Howard Hughes Medical Institute, University of Colorado Boulder, Boulder, Colorado 80303, USA; 4 Department of Molecular, Cellular and Developmental Biology, University of Colorado Boulder, Boulder, Colorado 80303, USA

**Keywords:** RNA, pUG-fold, DNMT1, epigenetics, G-quadruplex, DNA methylation

## Abstract

Aberrant DNA methylation is one of the earliest hallmarks of cancer. DNMT1 is responsible for methylating newly replicated DNA, but the precise regulation of DNMT1 to ensure faithful DNA methylation remains poorly understood. A link between RNA and chromatin-associated proteins has recently emerged, and several studies have shown that DNMT1 can be regulated by a variety of RNAs. In this study, we have confirmed that human DNMT1 indeed interacts with multiple RNAs, including its own nuclear mRNA. Unexpectedly, we found that DNMT1 exhibits a strong and specific affinity for GU-rich RNAs that form a pUG-fold, a noncanonical G-quadruplex. We find that pUG-fold-capable RNAs inhibit DNMT1 activity by inhibiting binding of hemimethylated DNA, and we additionally provide evidence for multiple RNA binding modes with DNMT1. Together, our data indicate that a human chromatin-associated protein binds to and is regulated by pUG-fold RNA.

## INTRODUCTION

Many chromatin-modifying enzymes that bind DNA and histones have also been found to bind RNA. This RNA binding has been proposed to play a role in both the recruitment and regulation of enzyme activity, but detailed mechanisms of RNA-mediated regulation remain poorly understood (Davidovich and Cech 2015; Beltran et al. 2019; Winkler and Dimitrova 2022). Examples of chromatin-associated proteins that exhibit both DNA and RNA binding include PRC2, WDR5, CTCF, RNA polymerase II, many transcription factors, and the DNMT family of methyltransferases (Di Ruscio et al. 2013; Ponicsan et al. 2013; Merry et al. 2015; Wang et al. 2015, 2017; Long et al. 2017, 2020; Saldaña-Meyer et al. 2019; Steiner et al. 2022).

The DNA methyltransferases methylate cytosines within CpG dinucleotides—a major mechanism of epigenetic gene repression (Bird 2002; Lyko 2018). CpG islands are prevalent near or in many promoters, thus the need for faithful methylation maintenance in these regions is critical (Esteller 2002). The failure to maintain the methylation state of the genome results in genomic instability and disease susceptibility (Feinberg and Vogelstein 1983; Baylin and Herman 2000; Eden et al. 2003). There are three classes of DNMTs; DNMT1 and DNMT3A/B are DNA methyltransferases while DNMT2 methylates tRNAs (Goll and Bestor 2005; Goll et al. 2006; Jeltsch et al. 2016; Lyko 2018). DNMT1 is a maintenance DNA methyltransferase that restores the methylation state to newly replicated hemimethylated DNA, and DNMT3A/B are de novo methyltransferases that deposit methyl marks during development and differentiation (Gruenbaum et al. 1982; Jeltsch 2006; Ren et al. 2018b; Gao et al. 2020). While these distinctions are generally true, there has been evidence of de novo activity by DNMT1 as well (Wang et al. 2020; Haggerty et al. 2021). DNMT1 has been shown to interact with, and be regulated by, a variety of cofactors that include other proteins, small molecules and RNA (Estève et al. 2006; Di Ruscio et al. 2013; Merry et al. 2015; Hendrickson et al. 2016; Ren et al. 2018a, 2021).

DNMT1 has been demonstrated to directly associate with a variety of RNAs, which seem to have varying effects on DNMT1 activity and localization. DACOR1, a long noncoding RNA (lncRNA) which is down-regulated in colon cancer, was shown to interact with DNMT1 (Merry et al. 2015). Loss of this interaction promoted global hypomethylation, although the precise mode of interaction between this RNA and DNMT1 remains unclear (Merry et al. 2015; Somasundaram et al. 2018). Additionally, DNMT1 has been shown to directly interact with the lncRNA ecCEBPA, an antisense lncRNA originating from the *CEBPA* locus (Di Ruscio et al. 2013). DNMT1 is a large protein (183 kDa) with a large regulatory N-terminal region and a catalytic C-terminal domain. ecCEBPA RNA was shown to bind to the C-terminal region of DNMT1 and promote local hypomethylation at its native locus by preventing DNMT1 from binding the promoter, thereby allowing for transcription of the *CEBPA* gene. In the same study, genome-wide analysis indicated that inhibition of DNMT1 action by nascent RNA was general beyond the *CEBPA* locus.

Additionally, DNMT1 has been shown to be inhibited by binding to various miRNAs, including miR-155-5p (Zhang et al. 2015). This RNA interacts with the C-terminal region of the protein and was shown to be inhibitory to DNA binding and therefore methyltransferase activity. This inhibition was seen with various RNA constructs, including RNAs with G-quadruplex-forming potential.

In this study, we investigate the sequence and structure specificity of DNMT1-RNA interactions and the effect of RNA binding on methyltransferase activity. We first use formaldehyde RNA immunoprecipitation, followed by next-generation sequencing (fRIP-seq) on endogenous DNMT1 in two different cell lines, and find that DNMT1 interacts with many RNAs, and surprisingly, its own mRNA. Upon further analysis of RNA sequence and structure preference of DNMT1, we see a high and specific affinity for a recently discovered RNA structure called a poly-(UG)-fold (pUG-fold) (Roschdi et al. 2022). We also find that DNMT1 displays a general but lower affinity for a variety of other RNAs. Finally, we demonstrate that binding of pUG-fold RNA to DNMT1 inhibits DNMT1 methyltransferase activity.

## RESULTS

### DNMT1 binds to many different RNAs in the nucleus, including its own mRNA

**FIGURE 1.**
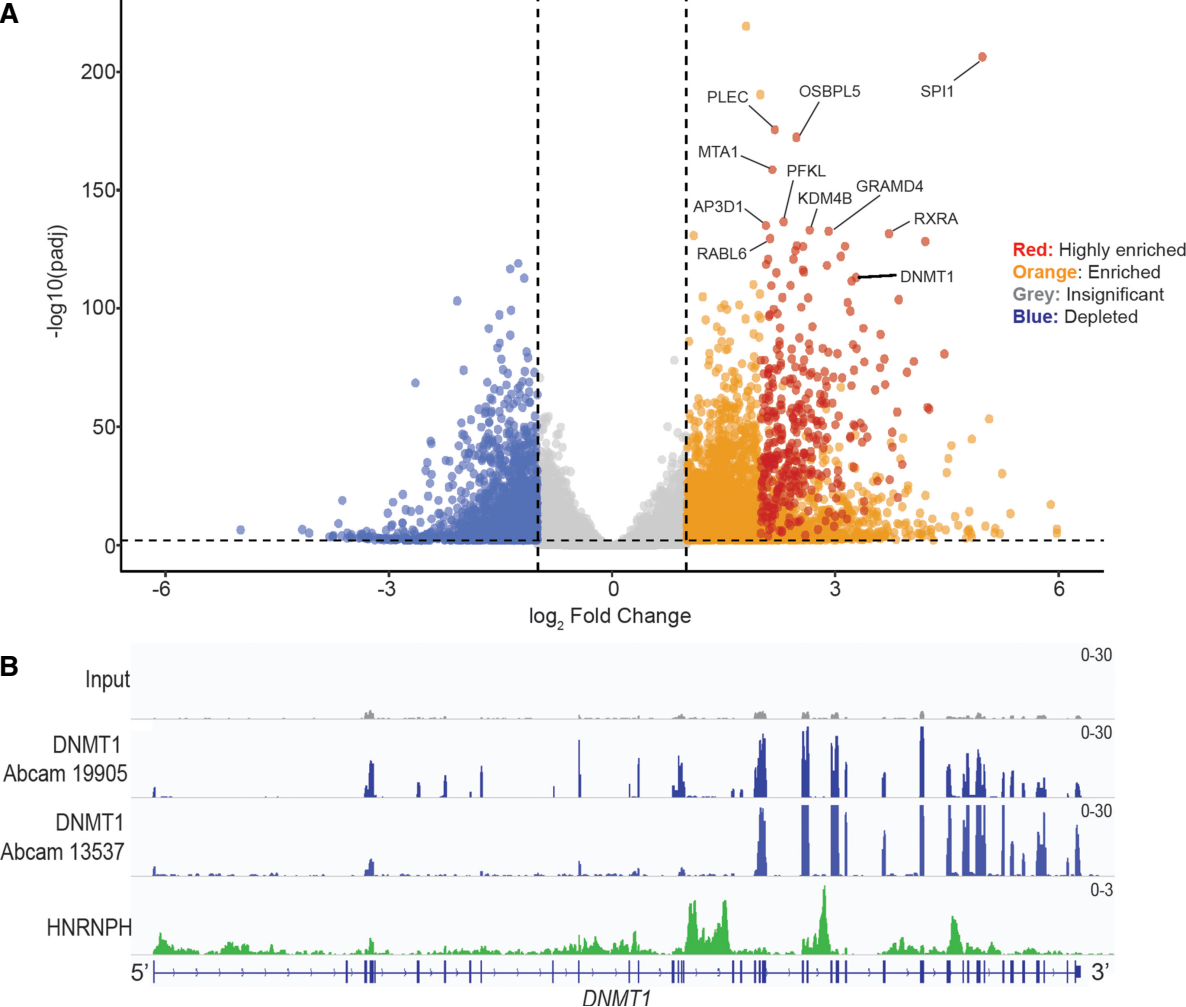
DNMT1 has numerous binding partners including its own mRNA. (*A*) A volcano plot showing enriched RNAs from fRIP-seq in K562 cells. Highly enriched RNAs (red) have been classified by a Log_2_FoldChange > 2, baseMean > 100, and adjusted *P*-value (*P*_adj_) < 0.001. Enriched RNAs (orange) are classified by a Log_2_FoldChange > 1, baseMean > 100, and P_adj_ < 0.001. Nonsignificant and depleted RNAs are shown in gray and blue, respectively. The top 10 enriched RNAs (by P_adj_) are indicated. DNMT1 (22nd most enriched) is also indicated. (*B*) Integrated Genomics Viewer (IGV) track of DNMT1 (blue) and HnRNP H (green) fRIP peaks over the *DNMT1* mRNA.

To dissect the binding preference of DNMT1 to various RNAs, we performed DNMT1 fRIP-seq in two different human cell lines, the leukemia cell line K562 and induced pluripotent stem cells (iPSCs) (Fig. 1A; Supplemental Fig. S1A,B). Additionally, we performed fRIP-seq with two different DNMT1-targeting antibodies to identify consistent RNA binding events. There was good overlap between the data sets from both antibody pulldowns in both cell types (Supplemental Fig. S1C,D). DNMT1 bound about 3300 different RNAs in K562 cells and about 1200 in iPSCs [Log_2_FoldChange > 1, Adjusted *P*-value (*P*_adj_) < 0.01]; this difference correlates with the higher level of DNMT1 in the K562 cell line as measured by western blot signal intensity (Fig. 1A; Supplemental Fig. S1A,B). Most of the significantly enriched RNAs were mRNAs, and one of the most consistently enriched RNAs, regardless of cell line or antibody, was the *DNMT1* mRNA (Fig. 1A; Supplemental Fig. S1B).

The highest percentage of DNMT1 fRIP-seq peaks was within introns and regulatory regions (Supplemental Fig. S2A). However, the interaction between DNMT1 and its mRNA was biased to the fully spliced and processed mRNA as evidenced by peaks observed mainly over exonic regions (Fig. 1B). To check if our fRIP-seq assay was biasing the observed results, we used HnRNP H, a known intronic binding protein, as a control and saw peaks mainly within introns of the same mRNA (Fig. 1B; Supplemental Fig. S2A). Because DNMT1 is a nuclear protein, its preference for binding to the fully spliced transcript was unexpected. To test if the observed interaction between DNMT1 and its mRNA could be due to crosslinking of the nascent DNMT1 peptide and the mRNA in the cytoplasm during translation, we performed the same fRIP-seq experiments after nuclear fractionation (Supplemental Fig. S2B). We observed the same DNMT1–mRNA interaction in the nucleus, thereby supporting the hypothesis that this interaction is a nuclear binding event (Supplemental Fig. S2C). In addition to binding to the fully spliced transcript, we noticed a preference for binding toward the 3′ end of the mRNA. Although there was a slight PCR amplification bias toward the 3′ end of the *DNMT1* mRNA, it was not enough to explain the observed 3′-binding preference of DNMT1 (Supplemental Fig. S2D).

### DNMT1 shows binding preference for GU-repeat containing RNA

**FIGURE 2.**
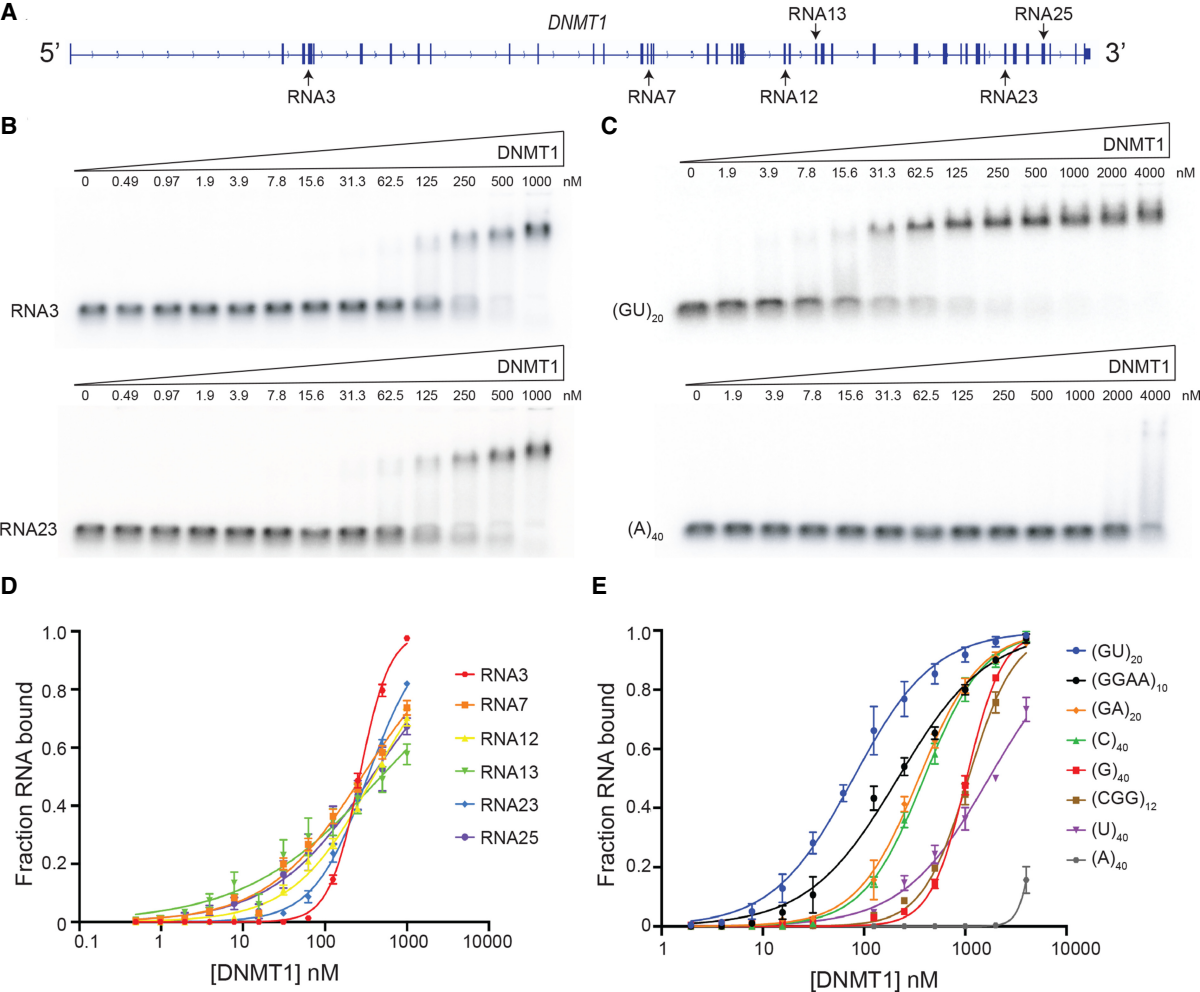
DNMT1 binds to a variety of RNAs with different affinities in vitro. (*A*) Schematic of the location of in vitro transcribed RNAs. (*B*) Representative EMSAs with radiolabeled trace amounts of RNA3 and RNA23 with increasing amounts of DNMT1 protein. (*C*) Representative EMSAs with radiolabeled trace amounts of (GU)_20_ and (A)_40_ with increasing concentrations of DNMT1. (*D*) Binding curves for all *DNMT1* mRNA constructs. (*E*) Binding curves for all synthetic 40-mer RNA oligonucleotides. In *D* and *E*, points represent mean values and error bars represent SD for *n* = 3 replicates.

To further test the affinity of DNMT1 to its own mRNA, we made six ∼200 nt RNA truncations by in vitro transcription (Fig. 2A; Supplemental Table 1). These RNAs span the regions that show peaks in the fRIP-seq of Figure 1B (Fig. 2A). To analyze binding of these RNAs, we performed electrophoretic mobility shift assays (EMSAs) with recombinant full-length human DNMT1 (Supplemental Fig. S3A,B). While all RNAs bound with similar affinities, RNA3 had the highest affinity (∼250 nM *K*_d_^app^) (Table 1; Fig. 2B,D). Several of the RNAs (# 7, 12, 13, 25) had broad binding curves, which we interpret to indicate that they fold into two or more conformers that have different affinities for DNMT1. This notion is further supported by the presence of multiple bands for these RNAs when run on a native gel (Supplemental Fig. S3C). In general, the affinity of DNMT1 for the various mRNA truncations is similar regardless of sequence composition or predicted amount of secondary structure, indicating that DNMT1 has a broad general affinity for RNA.

**TABLE 1.**
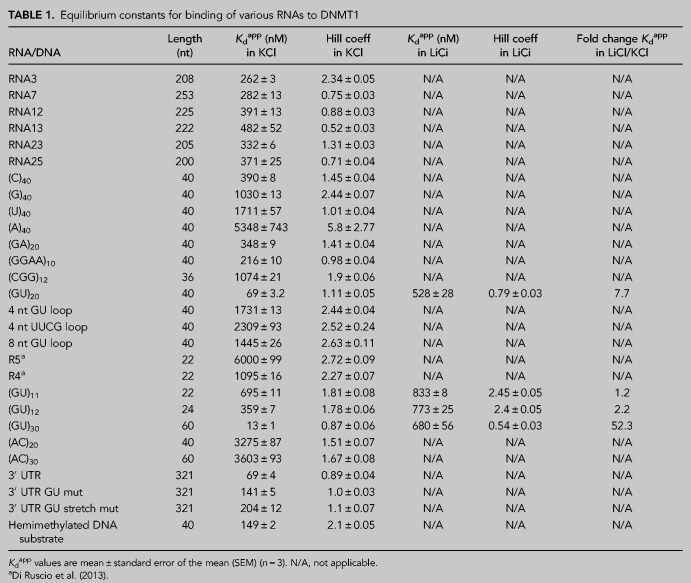
Equilibrium constants for binding of various RNAs to DNMT1

Given that the natural RNA sequences tested bound DNMT1 with similar affinities, we used a series of synthetic 40-mer RNAs with the aim of identifying any sequence specificity. Indeed, these binding assays revealed substantial specificity. Among the homopolymers, DNMT1 had the highest affinity for (C)_40_ and undetectable binding to (A)_40_ (Table 1; Fig. 2C,E). The G4 RNA (GGAA)_10_ bound with somewhat higher affinity than (GA)_20_, which has the same base composition but cannot form G4s. The observed higher affinity for G4s is consistent with previous studies showing direct interactions of DNMT1 with both RNA and DNA G4s (Zhang et al. 2015; Mao et al. 2018). Additionally, we found that DNMT1 bound to many of these RNAs with equal or higher affinity than to a hemimethylated DNA substrate of equal length (Table 1). Interestingly, DNMT1 showed particularly high affinity to (GU)_20_ (69 nM *K*_d_^app^) (Table 1; Fig. 2C,E). Intrigued by this result, we further investigated the nature of DNMT1's affinity for GU repeats.

### DNMT1 interacts with RNAs capable of forming noncanonical G4s

Since G and U can form wobble base pairs, it seemed possible that DNMT1 might be recognizing a stem–loop structure with G-U pairs in the stem and a hairpin loop comprised of GU repeats. We therefore designed hairpins with different sequence compositions and loop sizes, and we added GU repeats within the loops to tease apart potential binding preferences of the loop versus stem (Supplemental Fig. S4A; Supplemental Table 1). All stem–loop constructs ran with much higher mobility on a native gel compared to (GU)_20_, indicating that (GU)_20_ does not in fact form a stem–loop structure under these conditions (Supplemental Fig. S4B). This idea was further supported by the fact that while DNMT1 bound to all these stem–loop structures, it had very low affinity, *K*_d_^app^ > 1 µM (Table 1; Supplemental Fig. S4C). In addition, a 22 nt stem–loop RNA (R5) that was previously shown to bind to DNMT1 (Di Ruscio et al. 2013) bound with low affinity (>1 µM *K*_d_^app^) in our conditions (Table 1; Supplemental Table 1).

**FIGURE 3.**
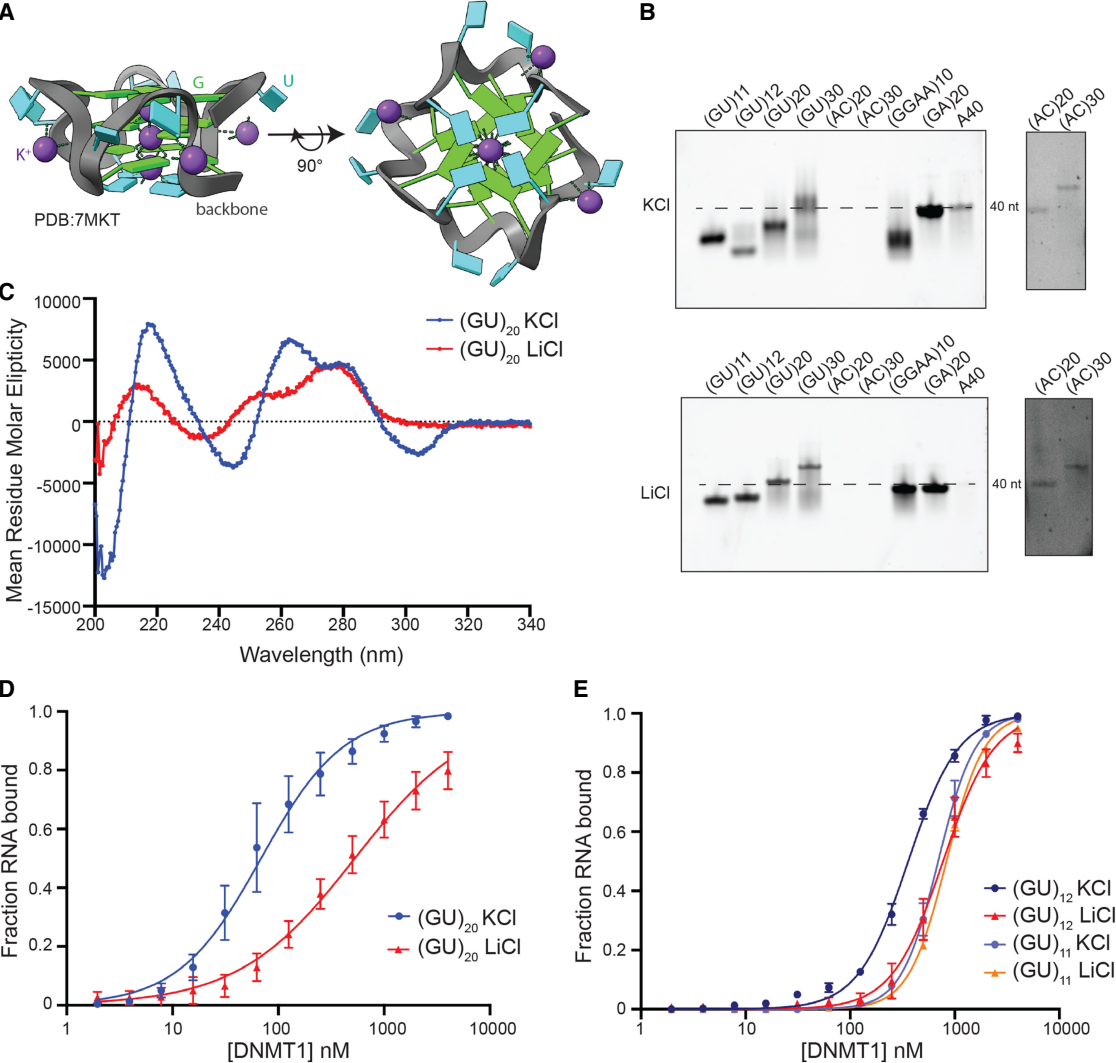
DNMT1 binds with high affinity to pUG-fold RNA. (*A*) Schematic of a pUG-fold. G and U residues are labeled in green and cyan, respectively. The phosphate backbone is highlighted in gray, and coordinated K^+^ ions are indicated by purple spheres. PDB: 7MKT. (*B*) Native gel of indicated RNAs with either 100 mM KCl or LiCl included in the folding buffer, the gel running buffer, and the gel itself. (A)_40_ was used as a marker for an unstructured single-stranded RNA (dashed line). Longer exposure of (AC)_20_ and (AC)_30_ staining is shown to the *right*, due to the low staining of AC sequences by SybrGold. (*C*) Circular dichroism spectra of (GU)_20_ in 100 mM KCl (blue) and LiCl (red). (*D*) Binding curves of (GU)_20_ bound to DNMT1 in 100 mM KCl (blue) and LiCl (red). (*E*) Binding curves of (GU)_12_ and (GU)_11_ bound to DNMT1 in 100 mM KCl (blue and light purple, respectively) and LiCl (red and orange, respectively). In *D* and *E*, points represent mean values and error bars represent SD for *n* = 3 replicates.

Recently, another study demonstrated that GU repeats can fold into a structure termed a pUG-fold (Roschdi et al. 2022). pUG-folds are noncanonical parallel G4 structures with a left-handed backbone in which Us are looped out to allow the formation of three stacked G-quartets capped by a terminal U-quartet. As with canonical G4s, the pUG-fold requires K^+^ ions in order to fold stably (Fig. 3A). To test for a pUG-fold structure in (GU)_20_, we first ran native gels in K^+^ versus Li^+^ to determine if its folding was K^+^-dependent. Indeed, (GU)_20_ ran with much faster mobility in K^+^ compared to Li^+^, indicating it is forming a K^+^-dependent compact structure (Fig. 3B). Furthermore, circular dichroism of (GU)_20_ gave a distinct G4-like spectrum in K^+^ but not in Li^+^ (Fig. 3C). Together, these experiments demonstrate that (GU)_20_ is capable of forming a pUG-fold.

Next, we tested the affinity of DNMT1 for (GU)_20_ in K^+^ versus Li^+^ to determine if the pUG-fold was necessary for DNMT1 binding. Indeed, (GU)_20_ binding affinity was substantially reduced (eightfold) in Li^+^ compared to K^+^ (Table 1; Fig. 3D). We conclude that DNMT1 binds both the pUG-folded GU repeats (K^+^) and unfolded GU repeats (Li^+^), with substantial preference for the folded form. Intriguingly, DNMT1 seems to exhibit a specific affinity for pUG-folds compared to canonical G4s, as indicated by the increased affinity for (GU)_20_ over RNAs of equal length that can form canonical G4s [threefold increase over (GGAA)_10_ and a >10-fold increase over (G)_40_ (see Fig. 2B)].

Additionally, Roschdi et al. found that 12 GU repeats are required to form a single pUG-fold, while 11 repeats are insufficient. We therefore tested the binding of DNMT1 to (GU)_12_ and (GU)_11_ in both K^+^ and Li^+^. As predicted by the pUG-fold hypothesis, (GU)_12_ in K^+^ had a significantly higher affinity for DNMT1 than (GU)_12_ in Li^+^ or (GU)_11_ in either K^+^ or Li^+^ (Fig. 3E). Importantly, the similar binding curve and affinity of DNMT1 for (GU)_11_ in both K^+^ and Li^+^ ensures that it is the pUG-folding capacity and not the presence of Li^+^ ions/absence of K^+^ ions that affect the binding to DNMT1.

### DNMT1 exhibits a length-dependent affinity for GU repeats

**FIGURE 4.**
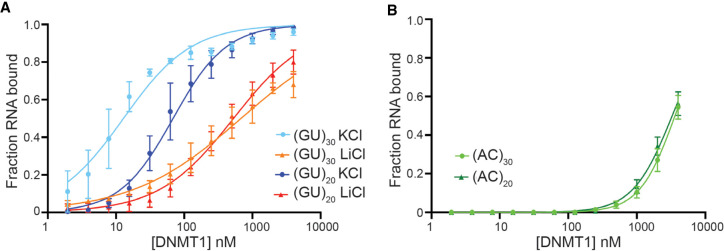
DNMT1 binds to GU repeats in a length-dependent manner. (*A*) Binding curves of (GU)_20_ in KCl and LiCl (blue and red, respectively) and (GU)_30_ in KCl and LiCl (light blue and orange, respectively). Corresponding EMSAs are shown in Supplemental Figure S5. (*B*) Binding curves of (AC)_20_ and (AC)_30_ with DNMT1 (dark green and light green, respectively). Corresponding EMSAs are shown in Supplemental Figure S5. In both panels, points represent mean values and error bars represent SD for *n* = 3 replicates.

Interestingly, (GU)_12_ has significantly reduced affinity (fivefold) compared to (GU)_20_, indicating that one pUG-fold alone does not confer very high affinity for DNMT1 (Fig. 3E). To determine if additional GU repeats stimulate tighter DNMT1 binding, we performed binding assays with (GU)_30_. As expected from the GU repeat length-dependent affinity observed in the previous experiment (Fig. 3E), DNMT1 showed greater than fourfold tighter binding to (GU)_30_ (*K*_d_^app^ = 13 nM) compared to (GU)_20_ (Table 1; Fig. 4A; Supplemental Fig. S5A,B). Interestingly, (GU)_30_ may be capable of forming two pUG-folds, or some other extended structure composed of G-quartets. To test if this is indeed the case, we repeated the same binding assay in Li^+^ and found a >50-fold reduction in affinity, arguing that the increased capacity to form pUG-folds or related structures indeed contributes greatly to DNMT1 binding affinity (Table 1; Fig. 4A; Supplemental Fig. S5A,B). To test if this GU repeat length–specific increase in binding affinity is specific to GU repeats, we performed the same experiment with (AC)_20_ and (AC)_30_. Notably, increased AC repeat content did not increase affinity, as seen with increasing GU repeats (Table 1; Fig. 4B; Supplemental Fig. S5C). Together, these results demonstrate that DNMT1 shows a high and specific affinity for pUG-fold RNA.

To further probe the structure of various pUG-fold RNAs, we performed circular dichroism (CD) and a thermal melting analysis with (GU)_12_, (GU)_20_, and (GU)_30_. Reduced CD signal for both (GU)_20_ and (GU)_30_, as well as increased absorbance at 260 nm, indicated that both RNAs have single-stranded GU sequences that reduce the overall pUG-fold signal, compared to (GU)_12_, which had a very strong pUG-fold signal (Supplemental Fig. S6A). All RNAs had identical spectra in LiCl, indicating that, regardless of length, each RNA forms a similar unstructured conformation in the absence of K^+^ ions. Furthermore, thermal melting analysis at 244 and 264 nm—the two wavelengths that are most indicative of the pUG-fold—demonstrated that all RNAs had similar two-step melting curves with the major melting transition around 55°C (Supplemental Fig. S6B,C); this is similar to the reported melting transition for a pUG-fold (Roschdi et al. 2022). Interestingly, the melting transition of (GU)_12_ in 100 mM KCl is about 20°C lower than that for TERRA, a canonical RNA G4 with the same number of nucleotides, indicating that pUG-fold G4s have reduced stability compared to canonical G4s (Wang et al. 2019).

### GU-repeat containing RNAs inhibit DNMT1 activity

**FIGURE 5.**
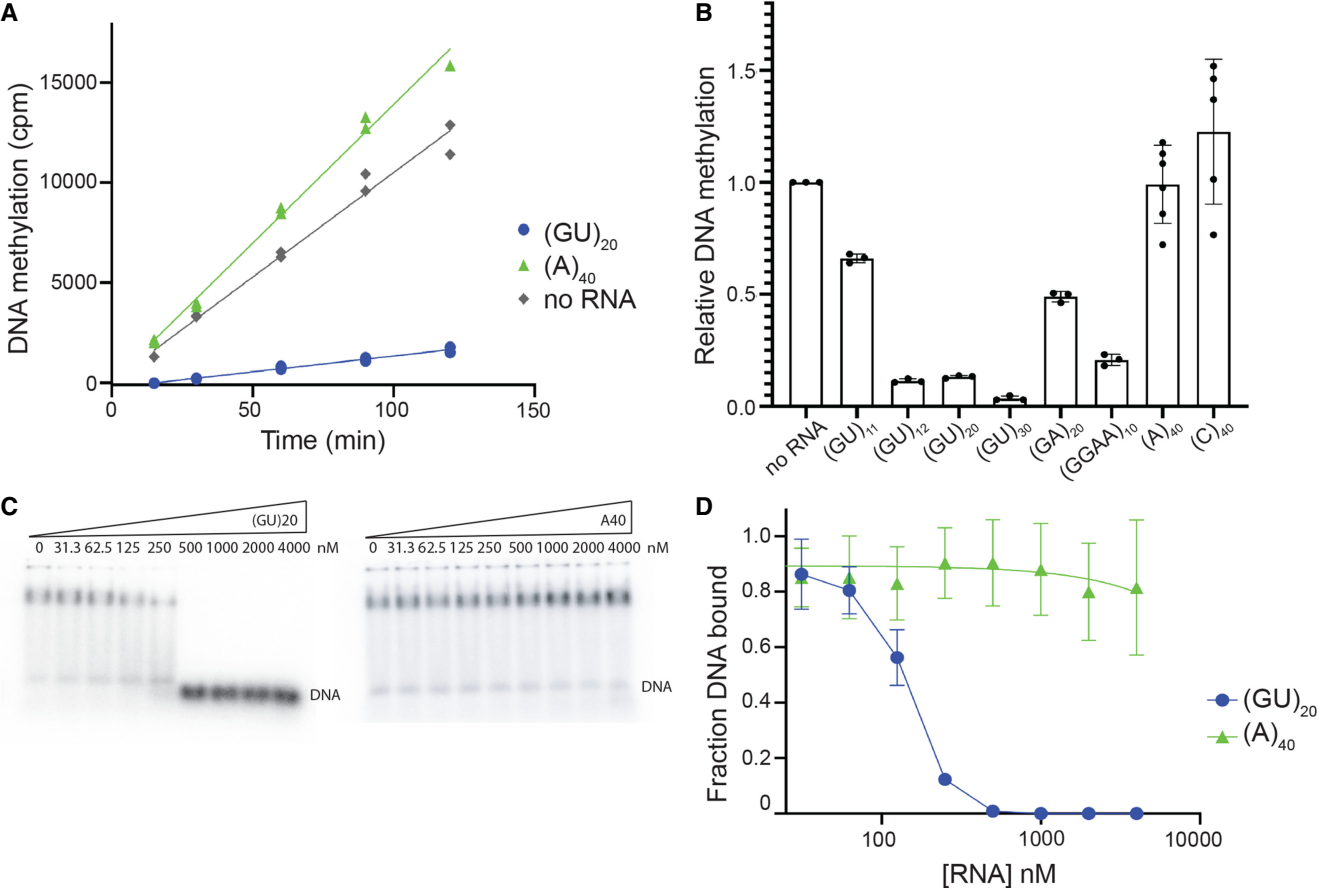
Differential inhibition of DNMT1 methyltransferase activity by different RNAs, with long GU repeats being particularly inhibitory. (*A*) Time course of DNA methylation with 0.25 µM DNMT1, 1 µM hemimethylated DNA substrate, and 1 µM (GU)_20_ or (A)_40_. DNA methylation was measured by incorporation of H^3^SAM and quantified by counts per minute (cpm). Points represent technical replicates. Entire experiment was repeated three times with similar results. (*B*) DNA methylation in the presence of 1 µM of each indicated RNA. Methylation levels have been normalized to amount of methylation measured in the “no RNA” sample. Points represent individual measurements, bars give mean value. The entire experiment was repeated four times with similar results, with the exception of (GA)_20_, which showed moderate inhibition in three out of four experiments and strong inhibition in one experiment. (*C*) Representative binding competition EMSAs with trace amounts of hemimethylated DNA substrate and 0.5 µM DNMT1 with increasing concentrations of (GU)_20_ and (A)_40_. (*D*) Binding curves for competition EMSAs shown in *C*, (GU)_20_ (blue) and (A)_40_ (green). Points represent mean values and error bars represent SD for *n* = 3 replicates. (A)_40_ data points are fit with a simple linear regression (green line).

Next, we determined the effect of pUG-fold RNA-binding on DNMT1 enzymatic activity. We first compared the DNMT1 methyltransferase activity on a hemimethylated DNA substrate in the presence of (GU)_20_ or (A)_40_ in physiologically relevant KCl concentrations (100 mM). Interestingly, 1 µM (GU)_20_ mostly abolished activity while (A)_40_ had no effect at the same concentration (Fig. 5A). We next looked at inhibition by other previously assayed RNAs. The inhibition by (GU)_30_ was stronger than that of (GU)_20_ (Fig. 5B; Supplemental Fig. S7), which is consistent with it having the highest binding affinity of all RNAs tested (Table 1). (GU)_12_, which is just long enough to form one pUG-fold, also strongly inhibited DNA methylation (Fig. 5B). In contrast, (GU)_11_, which is too short to form a pUG-fold, only mildly inhibited activity (Fig. 5B). These results indicate that pUG-fold RNA is inhibitory to DNMT1 enzymatic activity. Furthermore, a canonical G4 RNA (GGAA)_10_ was also inhibitory, although about twofold less than pUG-fold RNA (Fig. 5B). (GA)_20_, which has the same sequence composition as (GGAA)_10_ but no G4-potential, mildly inhibited activity of DNMT1. Like (A)_40_, (C)_40_ also showed no inhibition of DNMT1 activity, even though it has a much higher affinity for DNMT1 compared to (A)_40_ (Table 1). Together, these results argue that G4 RNA, specifically pUG-fold RNA, is particularly inhibitory to DNMT1 enzymatic activity.

To further understand the inhibition of DNMT1 activity, we measured the ability of cold (GU)_20_ and (A)_40_ to compete with a radiolabeled hemimethylated DNA substrate for binding. We saw strong competition for binding by (GU)_20_ with IC_50_ ∼ 100 nM (Fig. 5C,D; Supplemental Fig. S8A–D). These results indicate that pUG-fold RNA inhibits DNMT1 activity by preventing its hemimethylated DNA substrate from binding. Whether inhibition is due to direct competition in the active site or due to an allosteric change remains to be determined.

### DNMT1 binds to GU repeats within its own mRNA

**FIGURE 6.**
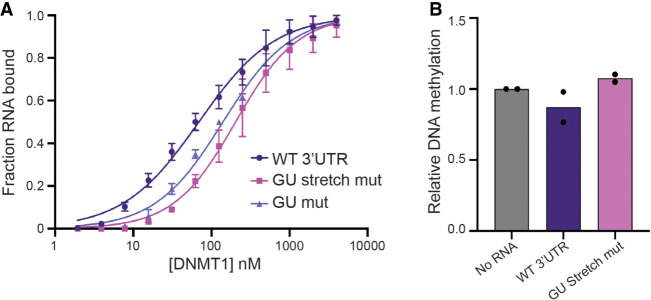
*DNMT1* 3′UTR binds to DNMT1 but does not inhibit activity in vitro. (*A*) Binding curves for the WT 3′UTR (purple) and GU mutant (light purple) and GU stretch mutant (pink). (*B*) Relative DNA methylation in the presence of 1 µM WT 3′UTR (purple) and GU stretch mutant (pink). Methylation levels have been normalized to amount of methylation measured in the “no RNA” (gray) sample. Bars give mean values, with individual data points shown as dots, *n* = 2.

Next, we sought to determine if the affinity of DNMT1 for GU-rich RNAs explains the binding to the *DNMT1* mRNA that we initially observed in the fRIP-seq results. We wondered if the bias in affinity toward the 3′ end of mRNA may be explained by high GU-repeat content. We analyzed the GU-content of the 3′-UTR of the *DNMT1* mRNA and noticed that it is indeed GU-rich with UG/GU being the most frequently occurring dinucleotide (20.8% GU/UG content) (Supplemental Fig. S9). To test if DNMT1 binds specifically to its own 3′UTR, and if this interaction is GU-repeat dependent, we first performed an EMSA with the full-length in vitro transcribed 321 nt 3′UTR (Fig. 6A). The 3′UTR had a binding affinity similar to that of (GU)_20_ (both 69 nM *K*_d_^app^), although the larger size of 3′UTR RNA may contribute to its affinity (Table 1). Mutation of all GU/UG-dinucleotides to AC/CA resulted in a twofold reduction in affinity (Fig. 6A). Furthermore, a more targeted mutation that removed only consecutive GU repeats of three or more gave a larger reduction in affinity (threefold), indicating that consecutive GU-repeat stretches contribute to binding to DNMT1 (Fig. 6A). The limited reduction in affinity is not surprising given DNMT1's general affinity for RNA (e.g., the *DNMT1* mRNA truncation RNA3, which is not very GU-rich).

We next tested if the WT 3′UTR or the GU-stretch mutant would influence DNMT1's methyltransferase activity. Neither RNA affected activity significantly in vitro (Fig. 6B). The high affinity of the 3′UTR RNA for DNMT1 and its lack of inhibition of activity is in striking contrast to the behavior of pUG-fold RNAs and indicates a different mode of binding.

## DISCUSSION

Here, we have uncovered two modes of RNA-binding by DNMT1: a high and specific affinity for pUG-fold RNA and a moderate affinity, seemingly promiscuous binding to many other types of RNA. The pUG-fold mode of binding inhibits DNA methyltransferase activity in an RNA concentration–dependent manner, while the more promiscuous RNA binding has little to no effect. Given the diversity of RNA sequences and structures bound by DNMT1, there may in fact be more than two modes of binding. The second mode of binding is consistent with previous studies showing that DNMT1 binds to multiple different RNAs of varying length and secondary structure with variable effects on DNMT1 enzymatic activity. (Di Ruscio et al. 2013; Merry et al. 2015; Zhang et al. 2015; Hendrickson et al. 2016). Additionally, we found that many RNAs bind DNMT1 with similar or higher affinity than that of a hemimethylated DNA substrate (Table 1). This preferred affinity for RNA over its natural DNA substrate has been observed previously as well (Di Ruscio et al. 2013).

pUG-folds have been shown to contribute to RNA silencing in *C. elegans* by recruiting the RNA-dependent RNA polymerase (RdRP) RRF-1 to synthesize siRNAs (Shukla et al. 2018; Preston et al. 2019; Roschdi et al. 2022). As there are no RdRPs in humans, the role of pUG-fold RNAs in humans is unclear, despite there being more than 20,000 pUG-fold sequences within the human transcriptome (Roschdi et al. 2022). To our knowledge, DNMT1 is the first human protein known to bind pUG-folds. It is interesting to note that while DNMT1 has never previously been shown to bind pUG-fold RNA, it has been shown to bind both canonical RNA and DNA G4s. RNA G4s have been shown to inhibit DNMT1 activity by directly competing with the DNA substrate, which now also includes pUG-fold RNA; in contrast, DNA G4s have been hypothesized to inhibit DNMT1 through allosteric mechanisms (Zhang et al. 2015; Mao et al. 2018). Furthermore, in our study we note that the strength of inhibition seems correlated with RNA structure rather than with affinity. RNAs with relatively high affinity, for example, 3′UTR, do not affect DNA methylation. On the other hand, RNAs with similar affinity [compare (C)_40_ and (GU)_12_ (Table 1)] have very different effects on activity, with (C)_40_ having no effect and (GU)_12_ being strongly inhibitory.

While noncanonical G4s have been characterized previously, especially in RNA aptamers, there is limited information regarding the affinity of RNA-binding proteins (RBPs) for canonical versus noncanonical G4s (Vasilyev et al. 2015; Yatime et al. 2015; Banco and Ferré-D'Amaré 2021). There is some evidence that the less stable nature of non-G quartets within noncanonical G4s may allow for a more seamless transition between G4 structures and other commonly found RNA secondary structures such as stem–loops (Warner et al. 2014). The increased affinity of DNMT1 for pUG-fold RNA over canonical G4 RNA in our study hints at a potential binding preference for such a hairpin-G4 structure over a G4 alone. Alternatively, it is possible that the increased affinity is driven by the pUG-fold-specific U-propeller loops or the terminally stacked U-quartet. It is also worth noting that natural RNAs with fewer than 12 consecutive UG repeats may still form pUG-folds, because the structure tolerates certain insertions and substitutions (Roschdi et al. 2022), similar to that of previously reported G4 structures (Lightfoot et al. 2019). A comprehensive analysis of how many and what types of insertions/substitutions are allowed is needed.

In this study, we found that DNMT1 binds to its own mRNA in two human cell types and that this interaction seems to be specific to the fully spliced transcript in the nucleus. Binding to a spliced transcript was unexpected, because on average DNMT1 fRIP signals for introns were higher than for exons, as expected for a nuclear protein (Supplemental Fig. S2A). Furthermore, DNMT1 binds with high affinity to its own 3′UTR but does not inhibit methyltransferase activity in vitro. This suggests that the DNMT1–*DNMT1* mRNA interaction could be regulatory in cells, for example, by influencing the binding of DNMT1 to its chromatin substrate or sequestering mature *DNMT1* mRNA in the nucleus, preventing it from being transported to the cytoplasm for translation. This could represent an autoregulatory mechanism to control DNMT1 levels in the cell.

Recent studies have demonstrated that binding of DNMT1 to a variety of ligands induces a conformational change in the enzyme (Ren et al. 2018a, 2021; Onoda et al. 2022). Additionally, the ATP-dependent methyltransferase DNMT5, a fungal relative of DNMT1, undergoes sequential conformational changes upon binding first to the cofactor SAM, then to ATP and finally to its native DNA substrate (Wang et al. 2022). It is interesting to consider the potential conformational changes that different types of RNA may stimulate when binding to DNMT1. For example, does (GU)_20_ simply occlude the active site when bound to prevent access to its native hemimethylated substrate? Or does it stimulate a conformational change that renders the active site unavailable? To our knowledge, there are no structural studies of DNMT1 bound to RNA. Future structural work on DNMT1/pUG-fold complexes are needed to better understand the nature of this intriguing interaction.

Formation of G4s within the UTRs of the mRNAs of many oncogenes and transcriptional regulators has been linked to translational repression (Kumari et al. 2007; Huppert et al. 2008; Shahid et al. 2010). Furthermore, disruption of RNA G4s within the 5′UTR of multiple oncogenes was shown to increase cancer susceptibility (Wolfe et al. 2014; Kosiol et al. 2021). It is interesting to consider that RNA G4s may play additional regulatory roles in trans through interactions with epigenetic regulators. Like DNMT1, PRC2—another chromatin-associated protein that promotes formation of heterochromatin—has been shown to bind and be regulated by G4 RNA (Kaneko et al. 2014; Long et al. 2017, 2020; Wang et al. 2017; Beltran et al. 2019). Curiously, the increased affinity of DNMT1 for pUG-fold G4s over canonical G4s is in contrast to the preferred affinity of PRC2 for canonical G4s versus GU-repeat RNA [greater than threefold increased affinity for canonical G4s (GGAA)_10_ versus (GU)_20_] (Wang et al. 2017). It is interesting to speculate that binding of G4s to chromatin-associated proteins may be an evolved mechanism for silencing of gene expression and that the difference in binding preference for different types of G4s could reflect a fine-tuning of RNA-based inhibition of different epigenetic factors.

## MATERIALS AND METHODS

### Cell culture and crosslinking

Ten million K562 cells (ATCC: CCL-243) or iPSCs (WTC-11) were grown for each experiment. Cells were spun down for 5 min at 500*g* and washed with 10 mL cold PBS. Cells were spun down and the supernatant was removed. Cells were fixed in 40 ml PBS + 0.1% formaldehyde at room temperature for 10 min. The formaldehyde solution was then quenched with 2 mL 2.5 M glycine (drops added slowly), followed by rotation at room temperature for 5 min. Cells were spun down at 500*g* for 5 min at 4°C. Supernatant was removed and cells were resuspended in 10 mL cold PBS + 1× Protease Inhibitor (Pierce Tablets). Cells were spun down for 5 min at 500*g* and supernatant was discarded. Pellets were resuspended in 10 mL ice-cold PBS + 1× PIC. Cells were spun down at 500*g* for 5 min and pellets were flash-frozen in liquid nitrogen and stored at −80°C or immediately used.

### fRIP

Crosslinked cell pellets were thawed and resuspended in 1 mL cold fRIP lysis buffer (50 mM Tris pH 8, 150 mM KCl, 0.1% SDS, 1% Triton-X, 5 mM EDTA, 0.5% sodium deoxycholate, 0.5 mM DTT, 1× Protease Inhibitor Cocktail [Roche], 100 U/mL RNasin Plus RNase Inhibitor [Promega]). Samples were rotated at 4°C for 10 min and then sonicated at 4°C in a Bioruptor UCD-200 (Diagenode) on the high setting using a 30 sec on/30 sec off cycle for 15 min. Cells were spun down at 13,000 RPM for 10 min at 4°C. Pellets were discarded, and 1 mL of cold fRIP wash buffer (25 mM Tris pH 7.5, 150 mM KCl, 5 mM EDTA, 0.5% NP-40, 0.5 mM DTT, 1× Protease Inhibitor Cocktail [Roche], 100 U/mL RNasin Plus RNase Inhibitor [Promega]) was added to 1 mL of supernatant to bring volume to 2 mL. Sample was filtered through a 0.45-micron filter into a new 2-mL tube and sample was split evenly between two new tubes (each with ∼5 million cells worth of lysate).

Pierce Protein A/G Dynabeads (Thermo Scientific) were equilibrated with cold fRIP wash buffer (25 mM Tris pH 7.5, 150 mM KCl, 5 mM EDTA, 0.5% NP-40, 0.5 mM DTT, 1× Protease Inhibitor Cocktail [Roche], 100 U/mL RNasin Plus RNase Inhibitor [Promega]), and 25 µL beads were added to each tube of lysate. Tubes were rotated at 4°C for 30 min. Beads were pulled down with a magnetic rack and supernatant was transferred to a new tube (precleared lysate).

Two 25 µL aliquots of precleared lysate were taken here as input and western blot samples. Precleared lysates were flash-frozen in liquid nitrogen and stored at −80°C until further use or used directly for IP.

One tube of precleared lysate was thawed for each IP condition. An amount of 6 µg of appropriate antibody was added to the lysate (DNMT1: Abcam 19905 lot no. GR3271225, Abcam 13537 lot no. GR3184365, HNRNPH: Bethyl A300-511A, lot no. 2). Tubes were vortexed gently and then rotated at 4°C for 2 h. An amount of 50 µL of equilibrated protein A/G magnetic beads was added to each tube of lysate. Samples were incubated for 1 h at 4°C while rotating. Beads were pulled and supernatant discarded. Beads were washed four times in 1 mL fRIP wash buffer (25 mM Tris pH 7.5, 150 mM KCl, 5 mM EDTA, 0.5% NP-40, 0.5 mM DTT, 1× Protease Inhibitor Cocktail [Roche], 100 U/mL RNasin Plus RNase Inhibitor [Promega]), each time rotating at 4°C for 10 min. Before removing the last wash, 100 µL of beads were transferred to a new tube. This 10% aliquot served as an IP sample for a western blot, and the remaining 90% was used for isolating RNA. Beads were pulled down for both the 900 and 100 µL samples and supernatant removed. Beads were either stored at −20°C or continued with for reverse crosslinking. The input and IP samples were thawed and 31 µL water was added to the input, and 56 µL water was used to resuspend the IP beads. To each sample, 33 µL 3× RCL buffer (3× PBS, 6% N-lauroyl sarcosine, 30 mM EDTA, 15 mM DTT), 0.2 mg Proteinase K (Invitrogen) and 1 µL RNasin Plus RNase Inhibitor (Promega) were added. Samples were incubated at 42°C for 1 h and then at 55°C for 1 h. RNA was isolated via phenol/chloroform extraction followed by ethanol precipitation. Each pellet was resuspended in 15 µL nuclease-free water.

### Library construction and next-generation sequencing

Purified RNA from input and IP samples were reverse transcribed, PCR amplified and barcoded using the KAPA RNA HyperPrep Kit with RiboErase. The resulting cDNA libraries were paired-end sequenced on an Illumina NextSeq 500 at a depth of 20–40 million reads/sample. All whole-cell fRIP experiments had three replicates and the nuclear fRIP had four replicates.

### fRIP-seq computational analysis

Read mapping and QC of sequenced samples was handled by nf-core/chipseq v1.1.0 pipeline (Ewels et al. 2020). Reads were mapped to hg38 (Gencode v32 annotation) using bwa and the default nf-core parameters. For gene level enrichment analysis, counts were quantified using Rsubread featureCounts and differential enrichment was calculated with DESeq2. All code to reproduce this analysis is available in the git repository: https://github.com/ljansson/dnmt1.git.

### Nuclear fractionation

Protocol was adapted from the Bönisch lab (Arrigoni et al. 2016). Twenty million K562 cells were grown/experiment. Cells were crosslinked and pelleted as described for the whole-cell fRIP above. Crosslinked pellets were thawed and gently resuspended in 1 mL cold Farnham lab (FL) buffer (5mM PIPES pH 8.0, 85 mM KCl, 0.5% NP-40, 1× Protease Inhibitor Cocktail [Roche]). Cells were then sonicated in a Covaris Ultrasonicator for 30 sec with 75W peak power, 2% duty factor and 200 cycles/burst at 4°C–10°C.

Cells were spun down at 1000*g* for 5 min at 4°C. Supernatant was removed and each nuclear pellet was washed once with 1 mL 1× FL buffer (5 mM PIPES pH 8.0, 85 mM KCl, 0.5% NP-40, 1× Protease Inhibitor Cocktail [Roche]). Pellets were spun down at 1000*g* for 5 min at 4°C. Supernatant was removed and pellets were resuspended in 500 µL of 1× fRIP lysis buffer (50 mM Tris pH 8, 150 mM KCl, 0.1% SDS, 1% Triton-X, 5 mM EDTA, 0.5% sodium deoxycholate, 0.5 mM DTT, 1× Protease Inhibitor Cocktail [Roche], 100 U/mL RNasin Plus RNase Inhibitor [Promega]). fRIP was then performed as described above for whole-cell pellets. Nuclear fractionation was confirmed by western blot. Antibodies used for western blot include: DNMT1 (Abcam 19905), EZH2 (Cell Signaling Technologies 5246S), and β-actin (Sigma-Aldrich A5441).

### Full-length human MBP-DNMT1 expression

Sf9 cells were used to generate baculovirus stocks using the Bac-to-Bac system (Life Technologies), according to the manufacturer's instructions. A baculovirus stock carrying the gene for DNMT1 was used to infect 4–12 L of Sf9 cells at a density of 2 million cells/mL in Sf-900 III SFM (Invitrogen cat. # 12658–027). Multiple 1 L cell cultures in 4 L flasks (VWR cat. # 32645–044) were incubated for 72 h at 27°C, 130 revolutions per minute (rpm). Cells were harvested by centrifugation for 20 min at 2000 relative centrifugal force (RCF) (JLA-8.1, Beckman) at 4°C, then frozen in liquid nitrogen and stored at −80°C until protein purification.

### Full-length human MBP-DNMT1 purification

A DNMT1 cell pellet was weighed and 50 mL of lysis buffer (10 mM Tris-HCl pH 7.5, 250 mM NaCl, 0.5% NP40, 1 mM TCEP) was added to every 6 g of pellet. Pellet was gently resuspended, followed by incubation at 4°C while rotating for 15 min. Cells were spun down at 18,000 rpm for 30 min at 4°C. Pellet was discarded and supernatant was incubated with equilibrated amylose resin (NEB, E8021L) (1 mL resin/2 g pellet) for 90 min at 4°C while rotating. Sample was then washed in batch with first 10 cv lysis buffer, 16 cv high salt buffer (10 mM Tris-HCl pH 7.5, 500 mM NaCl, 1 mM TCEP), 16 cv low salt buffer (10 mM Tris-HCl pH 7.5, 150 mM NaCl, 1mM TCEP) with 5 min spins at 1000*g* between each wash. MBP-DNMT1 was eluted in low salt buffer + 10 mM maltose. Elution was concentrated and cleaved overnight with 1:50 w/w ratio PreScission Protease at 4°C. Following cleavage, DNMT1 was purified by ion-exchange chromatography on a HiTrap 5 ml Heparin HP column (Cytiva), and fractions were pooled and concentrated. The heparin-purified sample was then further purified by size-exclusion chromatography on a Sepharose 6 Increase 10/300 GL column (Cytiva). Fractions were pooled and concentrated and aliquots of pure DNMT1 were flash-frozen and stored at −80°C until use.

### In vitro transcription

PCR primers were designed to PCR amplify *DNMT1* mRNA fragments from a full-length human *DNMT1* cDNA plasmid (plasmid details available upon request). PCR products were purified using the E.Z.N.A. Cycle Pure Kit (Omega Bio-tek) and used as templates for T7 in vitro transcription. The in vitro transcription reaction was incubated overnight at room temperature (37°C for 3′-UTR constructs). The reaction then incubated with Turbo DNase for 15 min. The RNA was then isolated using phenol/chloroform extraction and ethanol precipitation followed by PAGE purification.

### Electrophoretic mobility shift assay

In vitro transcribed RNAs were CIP-treated before end-labeling. An amount of 50 pmol of in vitro transcribed or synthetic RNA was incubated with 10 units T4 PNK (NEB) and 2 µL γ-^32^P-ATP in 1× PNK buffer and incubated for 1 h at 37°C. Radiolabeled RNAs were purified using QuickSpin columns (Roche), and radioactivity was measured by a scintillation counter. End-labeled RNA was folded by first heating to 95°C for 5 min followed by snap cooling on ice for 2 min, and then incubated for 30 min at 37°C in 1× refold buffer (50 mM Tris-HCl pH 7.5, 100 mM KCl, 2.5 mM MgCl_2_, 0.1 mM ZnCl_2_, 2.0 mM BME, 0.05% NP40, 5% v/v glycerol). Each binding reaction contained the indicated amount of DNMT1 plus 1000 cpm of end-labeled RNA in binding buffer (50 mM Tris-HCl pH 7.5, 100 mM KCl, 2.5 mM MgCl_2_, 0.1 mM ZnCl_2_, 2.0 mM BME, 0.05% NP40, 5% v/v glycerol, 0.1 mg/mL BSA [NEB], 0.1 mg/mL baker's yeast tRNA [Sigma R5636]). Reactions were incubated for 1 h at 30°C. Samples were run on a 1% agarose gel in 1× TBE and run for 90 min at 4°C at 66 V. The gel was dried and exposed overnight followed by imaging on a Typhoon FLA 9500 scanner (GE). All EMSAs were performed in triplicate. EMSAs were quantified using either ImageQuant or GelAnalyzer 19.1, and binding curves were generated using Prism 9. 40-mer RNAs were ordered from Dharmacon.

### Binding competition assays

RNAs were folded by first heating to 95°C for 5 min followed by snap cooling on ice and then incubated for 30 min at 37°C in 1× refold buffer (50 mM Tris-HCl pH 7.5, 100 mM KCl, 2.5 mM MgCl_2_, 0.1 mM ZnCl_2_, 2.0 mM BME, 0.05% NP40, 5% v/v glycerol). Each binding reaction contained 0.5 µM DNMT1, 1000 cpm of end-labeled DNA and the indicated concentration of cold RNA in 1× binding buffer (50 mM Tris-HCl pH 7.5, 100 mM KCl, 2.5 mM MgCl_2_, 0.1 mM ZnCl_2_, 2.0 mM BME, 0.05% NP40, 5% v/v glycerol, 0.1 mg/mL BSA [NEB], 0.1 mg/mL baker's yeast tRNA [Sigma R5636]). DNA was directly diluted in 1× folding buffer before incubating with DNMT1 and cold RNA. Hemimethylated DNA was ordered preannealed from IDT: ([5′TA(5mC)GTATC(5mC)GTATC(5mC)GGTTA(5mC)GTATC(5mC)GAATC(5mC)GTAC(5mC)GT 3′/5′ ACGGTACGGATTCGGATACGTAACCGGATACGGATACGTA 3′]). CpG-flanking sequences were designed to promote ideal binding/activity by DNMT1 (Adam et al. 2020).

Reactions were incubated for 1 h at 30°C. Samples were run on a 1% agarose gel in 1× TBE and run for 90 min at 4°C at 66 V. The gel was dried and exposed overnight followed by imaging on a Typhoon FLA 9500 scanner (GE). All competition EMSAs were performed in triplicate. Binding competition assays were quantified using GelAnalyzer 19.1, and binding curves were generated using Prism 9. The amount of dissociated DNA was calculated by first subtracting the unbound DNA signal in the no RNA lane from all other lanes. Then, the fraction of bound DNA was calculated by dividing the signal from the bound band from total signal (bound plus unbound with smear in between both bands’ background subtracted).

### In vitro methylation assay

Single point biochemistry assays were performed by incubating 0.25 µM DNMT1, 1.0 µM hemimethylated DNA (same as used for binding competition EMSAs), 1.0 µM folded RNA in 1× binding buffer (50 mM Tris-HCl pH 7.5, 100 mM KCl, 2.5 mM MgCl_2_, 0.1 mM ZnCl_2_, 2.0 mM BME, 0.05% NP40, 5% v/v glycerol, 0.1 mg/mL BSA [NEB], 0.1 mg/mL baker's yeast tRNA [Sigma R5636]) and 1.0 µM [^3^H] SAM (82.3 Ci/mmol, PerkinElmer). First, buffer, DNA, and [^3^H] SAM were combined in a master mix and aliquoted to appropriate tubes, followed by addition of indicated RNA. The reaction was then started by addition of protein. This order-of-addition was designed to ensure that the SAM, which is dissolved in acid, was neutralized with buffer before the protein was added. Reactions were incubated for 2.5 h at 37°C. Reactions were cooled to 4°C for 30 sec, quenched with 1.2 mM unlabeled SAM (NEB) and blotted onto Hybond-XL membrane. Membranes were allowed to air dry, then were batch washed (8 mL wash buffer per membrane) with 3× 50 mM KH_2_PO_4_, 1× 80% ethanol, 1× 100% ethanol. Membranes were allowed to air dry, then were soaked in ScintiSafe Econo 1 Scintillation Cocktail. A Beckman LS 6500 Scintillation Counter was used to measure ^3^H incorporation.

The inhibition patterns of various RNAs were determined by incubating 0.25 µM DNMT1, 1 µM hemimethylated DNA, 1 µM [^3^H] SAM (82.3 Ci/mmol, PerkinElmer) and various amounts of refolded RNA in 1× binding buffer. The same incubation, quenching, blotting, and washing protocols were followed as described above. Plots were generated using Prism 9.

### Circular dichroism

RNAs at 0.2 mg/mL were heated at 95°C for 5 min and then snap cooled, followed by folding in 1× refold buffer containing either KCl or LiCl (50 mM Tris-HCl pH 7.5, 100 mM KCl/LiCl, 2.5 mM MgCl_2_, 0.1 mM ZnCl_2_, 2.0 mM BME, 0.05% NP40, 5% v/v glycerol) at 37°C for 30 min. An amount of 150 µL of sample was added to a 0.5 mm pathlength cuvette and loaded into a Chirascan Plus Circular Dichroism and Fluorescence Spectrometer (Applied Photophysics), and the CD spectrum was recorded. Either KCl buffer or LiCl buffer alone was used as a baseline control for each salt condition. Measurements were taken from 200–340 nm. Cuvettes were rinsed with 3× buffer between each sample. When switching between KCl buffer and LiCl buffer, the cuvette was additionally washed 2× with H_2_O and once with 100% ethanol. Baselines were subtracted from experiment spectra and circular dichroism was converted to mean residue molar ellipticity.

Thermal melting analysis was performed from 20°C–85°C at 244 and 264 nm at a rate of 1°C/min with a 20 nm step size. RNAs were prepared and folded as described above. The change in molar ellipticity [Δε(cm^2^/mmol)] was normalized by converting the absorbance values at 20°C to 1 (for 264 nm) or −1 (for 244 nm). Thermal melts were performed three independent times at 264 nm and twice at 244 nm with similar results each day. First derivative curves (dF/dT) were generated in Prism and curves were smoothed with a second order polynomial with 10 neighbors on each side.

### Native gel electrophoresis

RNAs were heated at 95°C for 5 min and then snap cooled followed by folding in 1× refold buffer containing either KCl or LiCl (50 mM Tris-HCl pH 7.5, 100 mM KCl/LiCl, 2.5 mM MgCl_2_, 0.1 mM ZnCl_2_, 2.0 mM BME, 0.05% NP40, 5% v/v glycerol) at 37°C for 30 min. RNAs were loaded onto a 10% Native PAGE gel containing either 100 mM KCl or LiCl in 0.5× TBE. Samples were run at 80 V for 1 h in 0.5× TBE buffer plus 100 mM KCl/LiCl at room temperature. Gels were post-stained with SybrGold for 15 min and imaged by a FluorChem R Imager (ProteinSimple). Folding of *DNMT1* mRNA in vitro transcription products was analyzed by loading onto a 6% PAGE gel without additional salt in gel and loading buffer.

## SUPPLEMENTAL MATERIAL

Supplemental material is available for this article.

## COMPETING INTEREST STATEMENT

T.R.C. is a scientific advisor for Storm Therapeutics, Eikon Therapeutics, and Somalogic, Inc.

## Supplementary Material

Supplemental Material

## Figures and Tables

**Linnea Jansson-Fritzberg:**
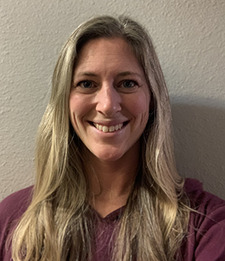


## MEET THE FIRST AUTHOR

***Meet the First Author(s)* is an editorial feature within *RNA*, in which the first author(s) of research-based papers in each issue have the opportunity to introduce themselves and their work to readers of *RNA* and the RNA research community. Linnea Jansson-Fritzberg is the first author of this paper, “ DNMT1 inhibition by pUG-fold quadruplex RNA.” Linnea is a postdoc in the Cech laboratory at the University of Colorado Boulder.**

**What are the major results described in your paper and how do they impact this branch of the field?**

We have uncovered that the DNA methyltransferase DNMT1 is inhibited by direct interaction with pUG-fold RNA. DNMT1 is an important epigenetic regulator that maintains the methylation state of the genome. DNMT1 is frequently misregulated in cancer, often resulting in aberrant expression of oncogenes and tumor suppressors. DNMT1 has been previously shown to interact with RNA, but the precise mode of interaction remains unclear. In this study we found that DNMT1 interacts with many different RNAs, including its own mRNA, but with especially high affinity and specificity to pUG-fold RNA; a noncanonical G-quadruplex structure composed of alternating Gs and Us. Additionally, this interaction inhibits the DNA methyltransferase activity of DNMT1 by preventing binding of the substrate DNA to the enzyme.

**What led you to study RNA or this aspect of RNA science?**

I joined a laboratory studying alternative splicing as an undergraduate at UC Santa Cruz and have loved learning about RNA ever since. I am fascinated by the complexity of RNA folding and the variety of functions that RNAs are involved in. I am particularly intrigued by the various roles of noncoding RNAs. When I joined the Cech laboratory, I decided to utilize the biochemical and biophysical expertise within the laboratory regarding how RNAs interact with epigenetic modifiers to spearhead a new project looking at the interplay between RNA and the DNA methyltransferase DNMT1.

**During the course of these experiments, were there any surprising results or particular difficulties that altered your thinking and subsequent focus?**

A particularly surprising result from this study was that consecutive GU-repeats form a quadruplex(G4)-like structure termed a pUG-fold. When we first started studying how DNMT1 interacts with RNA, we used a variety of synthetic RNA oligos that we had in the laboratory, including the 40-mer (GU)20. This RNA was originally designed to be a non-G4 control RNA, due to the common conception of intramolecular G4s requiring consecutive Gs. We noticed a strong binding affinity to this RNA but had no idea why. My initial hypothesis was that the Gs and Us form wobble base pairs within a hairpin, but when we tested the affinity of DNMT1 to other hairpins of equal length, it was nowhere near the affinity for (GU)20. Around this time, a colleague alerted me to a recent preprint by the Butcher laboratory at the University of Wisconsin Madison that found that consecutive GU-repeats form a noncanonical G4 structure termed a pUG-fold. When we saw this study, I had a lightbulb moment that this must be the structure that DNMT1 binds to. Subsequent experiments confirmed this hypothesis.

**What are some of the landmark moments that provoked your interest in science or your development as a scientist?**

My parents are both academic scientists, so I was exposed to science at an early age. By the time I went to college, I knew I wanted to work in a science-related field, but I had no experience working in a laboratory environment. When I had the opportunity to join a laboratory as an undergraduate researcher after my sophomore year, I immediately fell in love with laboratory work. Getting to witness laboratory members problem-solve and come up with experiments to test their hypotheses was something I couldn't wait to do myself. I eventually got a project of my own and loved that I had control of my day-to-day work and that I was working to better understand biology. From the time I joined the laboratory I knew I wanted to go to grad school and become a scientist. Additionally, while I majored in neuroscience, I discovered that my happy place was in molecular biology and biochemistry, fields that I eventually pursued during my PhD and postdoc.
