# Supplementary material for: DNMT1 inhibition by pUG-fold quadruplex RNA
Source: RNA. 2023 Mar;29(3):346–60. doi: 10.1261/rna.079479.122 (PMC9945446; doi:10.1261/rna.079479.122)
Supplement: Supplemental Material [file supp_29_3_346__DC1.html]

Supplemental Material 

# DNMT1 inhibition by pUG-fold quadruplex RNA

## Supplemental Material

- Supplemental\_material.pdf
