# Supplementary material for: DNMT1 inhibition by pUG-fold quadruplex RNA
Source: RNA. 2023 Mar;29(3):346–60. doi: 10.1261/rna.079479.122 (PMC9945446; doi:10.1261/rna.079479.122)
Supplement: Supplemental Material [file supp_079479.122_Supplemental_Material.pdf]

Supplemental information for

DNMT1 inhibition by pUG-fold quadruplex RNA

LINNEA I. JANSSON-FRITZBERG[1,2,4], CAMILA I. SOUSA[1,2,4], MICHAEL J. SMALLEGAN[1,3], JESSICA J. SONG[1,2], ANNE R. GOODING[4], VIGNESH KASINATH[2], JOHN L. RINN[1,2], THOMAS R. CECH[1,2,4]

[1]BioFrontiers Institute, University of Colorado Boulder, Boulder, CO, 80303, USA

[2]Department of Biochemistry, University of Colorado Boulder, Boulder, CO, 80303, USA

[3]Department of Molecular, Cellular and Developmental Biology, University of Colorado Boulder, Boulder, CO, 80303, USA

[4]Howard Hughes Medical Institute, University of Colorado Boulder, Boulder, CO, 80303, USA

[5]**Present address:** Department of Molecular and Cell Biology, University of California, Berkeley, Berkeley, CA 94720, USA

Corresponding author: thomas.cech@colorado.edu

Contents: **9 Supplemental Figures**
1 Supplemental Table

Supplemental Figures

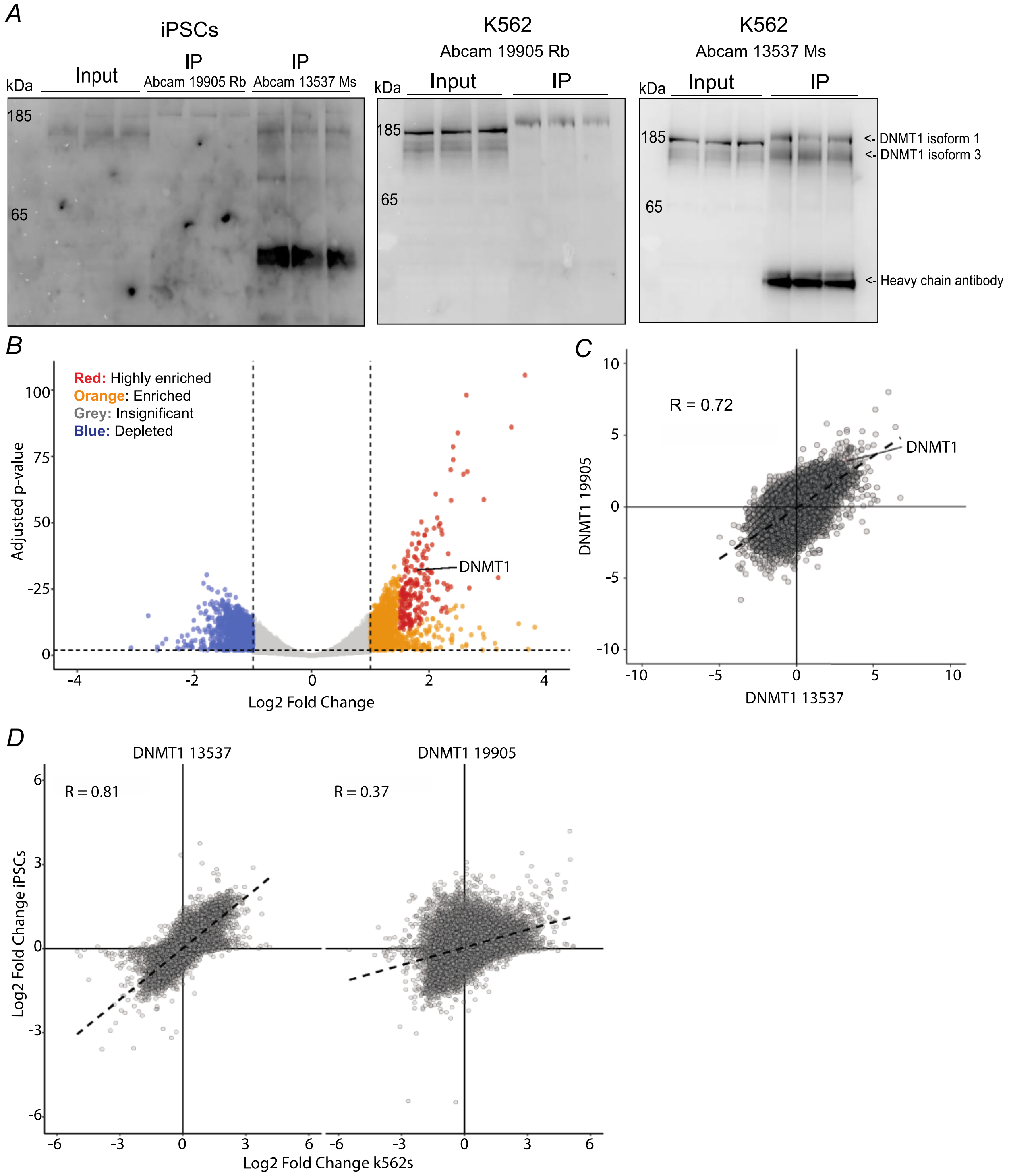

Supplemental Figure S1. DNMT1 fRIP in iPSCs and K562 cells. (*A*) Western blots of DNMT1 with indicated antibody in iPSCs (left) and K562 cells (two right blots). Abcam 13537 binds towards the C-terminal end of DNMT1 and therefore recognizes two isoforms of DNMT1 (isoform 1: 1-1616, isoform 3: 336-1616) while Abcam 19905 binds within the first 100 aa of

DNMT1 and therefore only recognizes the long (canonical) isoform 1. (*B*) Volcano plot of enriched RNAs in iPSCs. Highly enriched RNAs (red) have been classified by a Log2FoldChange > 1.5, baseMean > 100 and adjusted p-value (padj) < 0.001. Enriched are classified by a Log2FoldChange > 1, baseMean > 100 and padj < 0.001. Non-significant and depleted RNAs are shown in grey and blue, respectively. The location of the DNMT1 mRNA is indicated by an arrow. (*C*) Correlation between fRIPs using the two different DNMT1-targeting antibodies in K562 cells. The location of the DNMT1 mRNA is indicated by the arrow. The slope, R, of the correlation was calculated by linear regression. (*D*) Correlation between fRIPs in iPSCs vs K562s for the 13537 antibody (left panel) and 19905 antibody (right panel). The slope, R, of the correlation was calculated by linear regression. The lower correlation for the 19905 antibody is surprising and may reflect difference in abundance of enriched RNAs that bind only to the full-length DNMT1 isoform in the two different cell lines. Alternatively, the fRIP-seq in iPSCs was performed at a slightly higher read depth, which may also contribute to the observed difference between datasets.

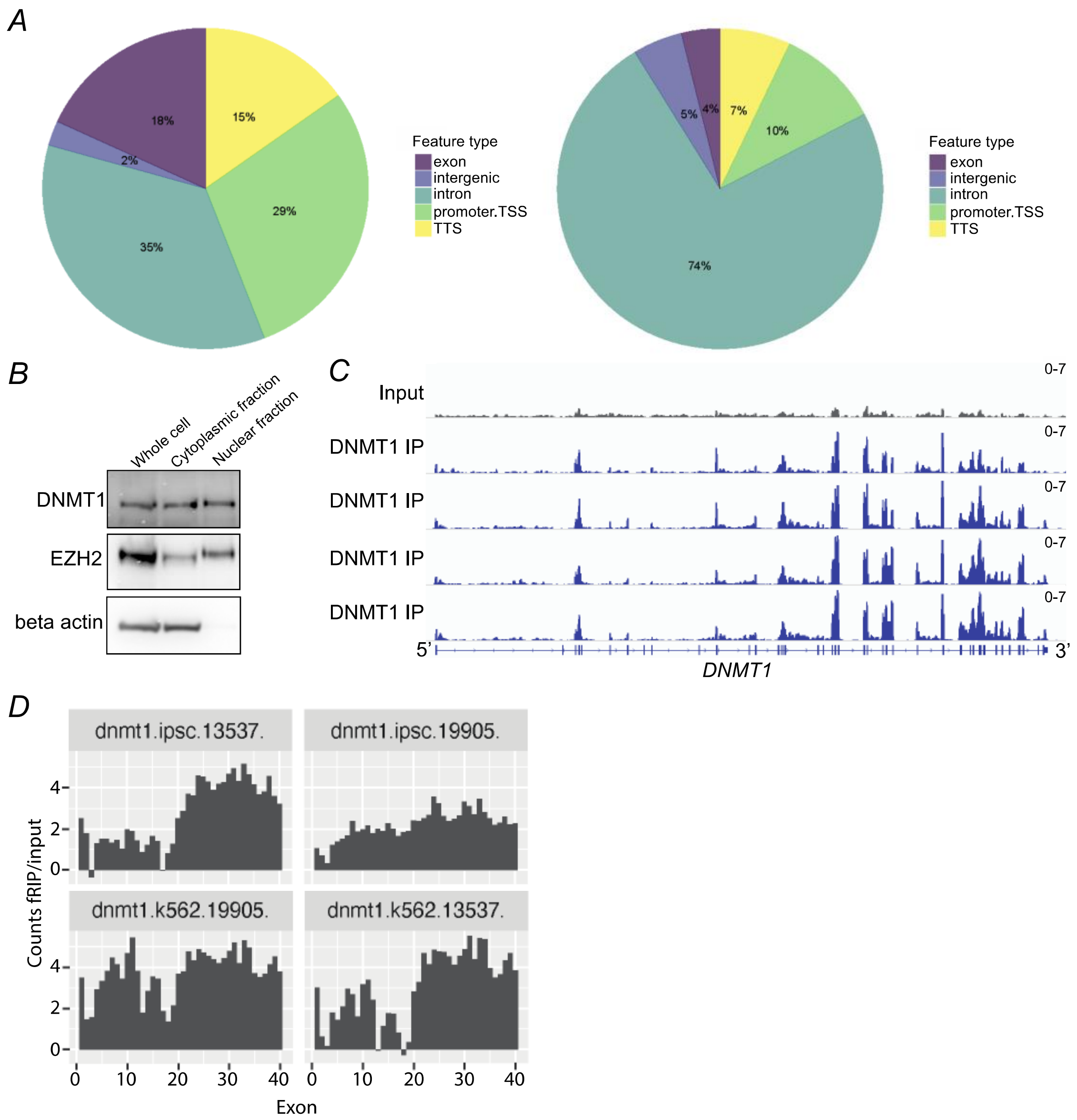

Supplemental Figure S2. DNMT1 interacts with its own mRNA in the nucleus.
(*A*) Percentage of peaks within each indicated feature type for DNMT1 (left) and HnRNP H (right) in K562 cells. Peak annotation was performed by HOMER. TSS: Transcription Start Site. TTS: Transcription Termination Site. (*B*) Western blot of nuclear fractionation shows that cytoplasmic proteins (β-actin) are not present in the nuclear fraction. EZH2 was used as a nuclear control protein. (*C*) IGV track of DNMT1 nuclear fRIP peaks (n=4) over the *DNMT1* mRNA. (*D*) Normalized counts per exon for each experiment was calculated by dividing the counts per exon in each fRIP by the counts per exon in the corresponding input.

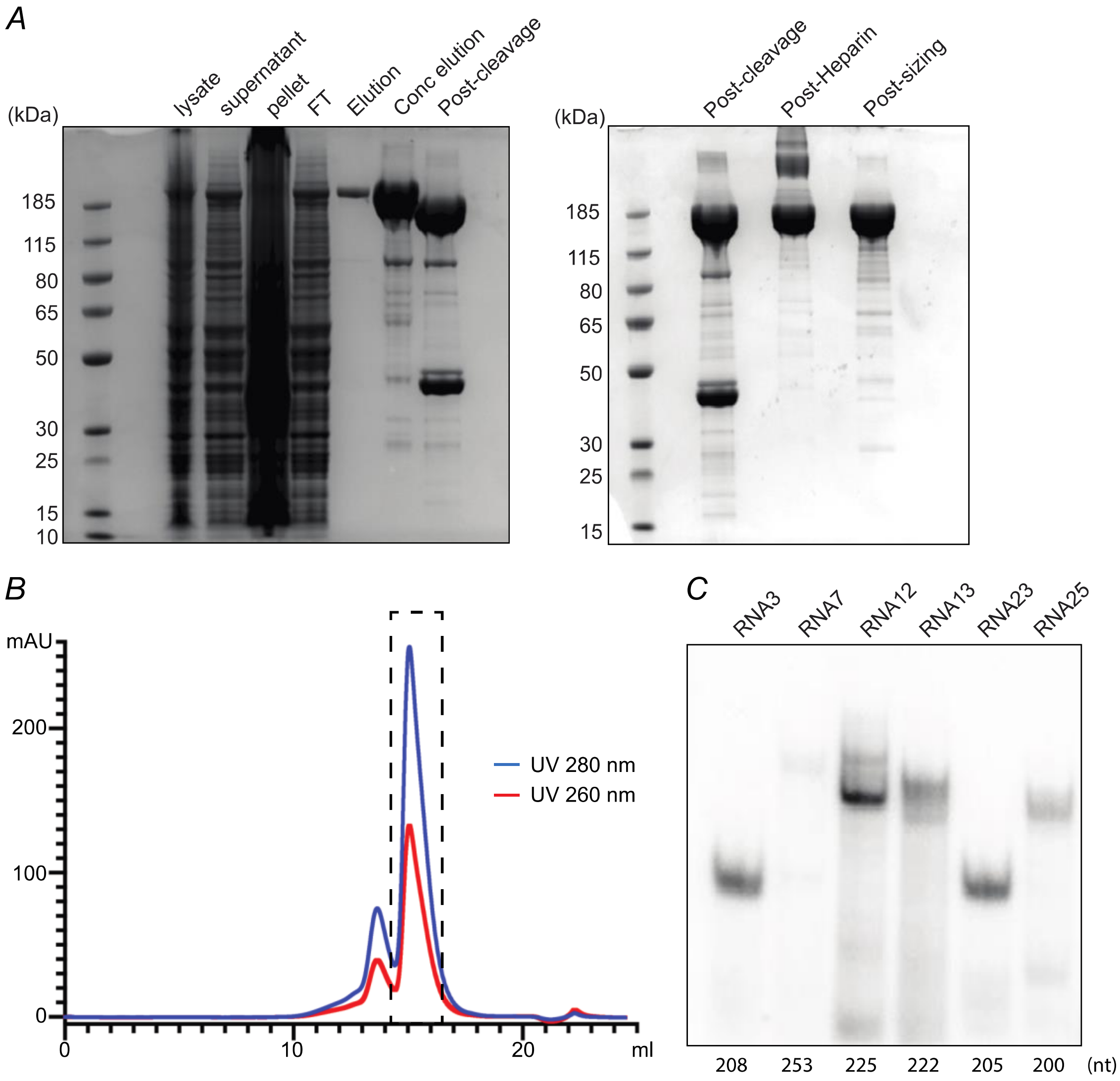

Supplemental Figure S3. Recombinant DNMT1 expression and in vitro transcribed RNAs. (*A*) Coomassie-stained gel of MBP-DNMT1 expression and purification (left panel) and DNMT1 purification after ion-exchange and size-exclusion chromatography (right panel). (*B*) Size exclusion chromatography trace of purified DNMT1. Dashed box indicates collected fractions. mAU – milli-absorbance unit. (*C*) 6% native polyacrylamide gel of in vitro transcribed RNAs. Size of each RNA is indicated at the bottom in nucleotides (nt).

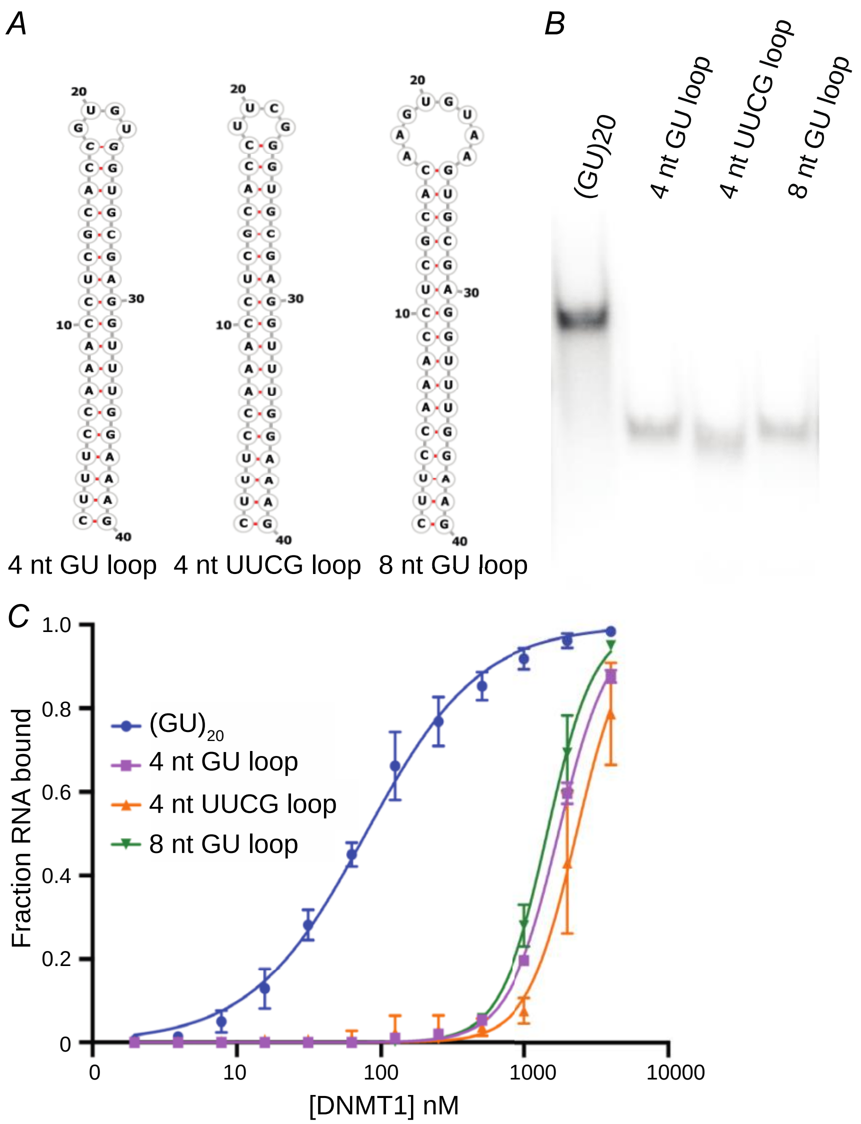

Supplemental Figure S4. DNMT1 binds RNA stem-loops with low affinity. (*A*) Predicted secondary structure of the three different stem-loop structures used in this study. All RNAs are 40 nt. The difference between the structures in the left vs. middle is GUGU in the loop vs. a stable UUCG tetraloop sequence, respectively (Hall 2015). The stem-loop at the right is similar to the structure at the left except it has a larger loop size. Secondary structure was predicted by RNAFold. (*B*) Native gel, without KCl in gel or running buffer, of $(GU)_{20}$ and the three different stem-loop constructs shown in *A*. (*C*) Binding curves of $(GU)_{20}$ and each RNA depicted in *A*. Points represent mean values, error bars give SD for n = 3.

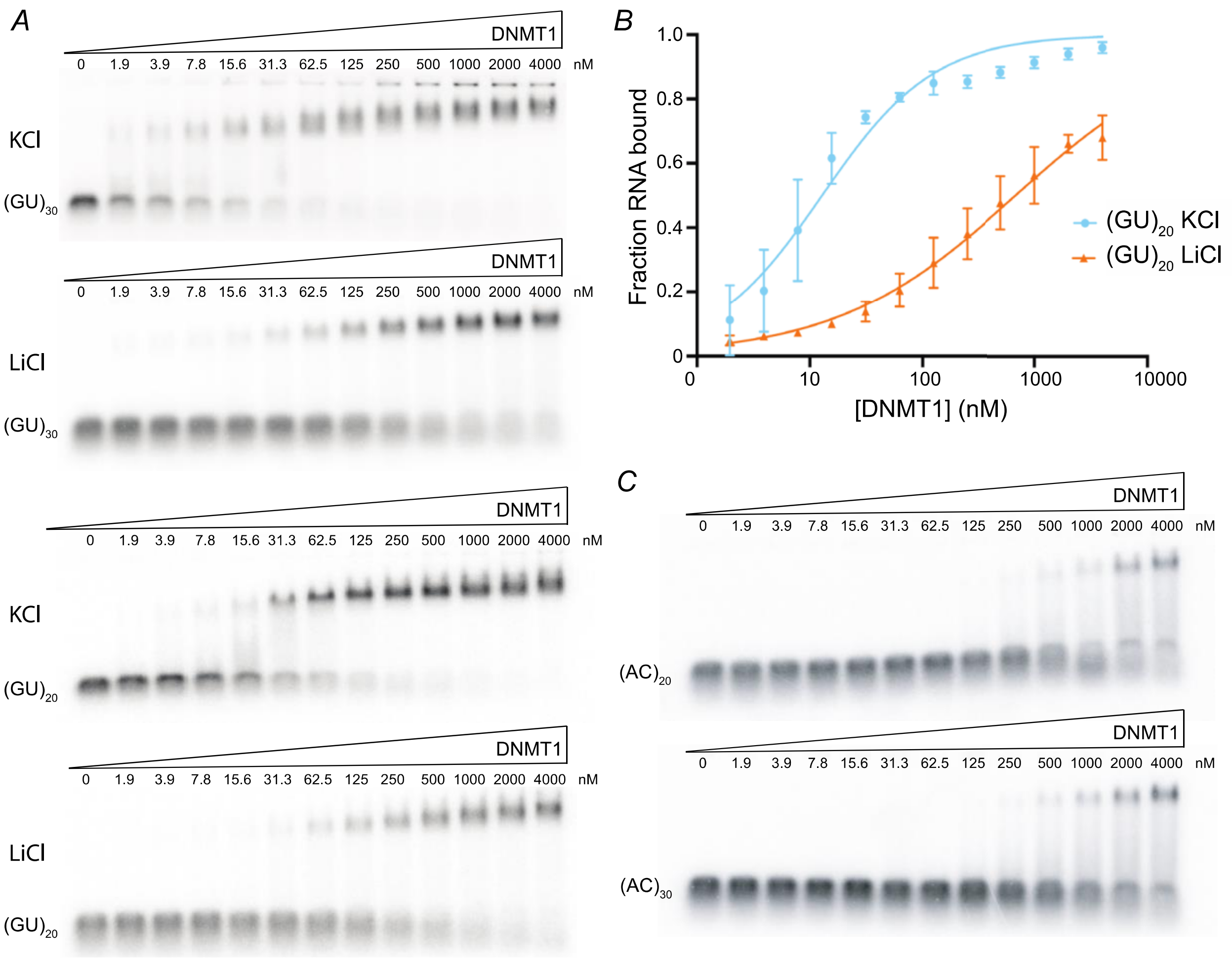

Supplemental Figure S5. DNMT1 binds to GU repeats in a length-dependent manner. (*A*) Representative EMSAs of $(GU)_{30}$ and $(GU)_{20}$ bound to DNMT1 in KCl and LiCl. (*B*) Binding curves of $(GU)_{30}$ in KCl (light blue) and LiCl (orange). Points give mean values, error bars give SD for n = 3. (*C*) Representative EMSAs of $(AC)_{20}$ and $(AC)_{30}$ bound to DNMT1.

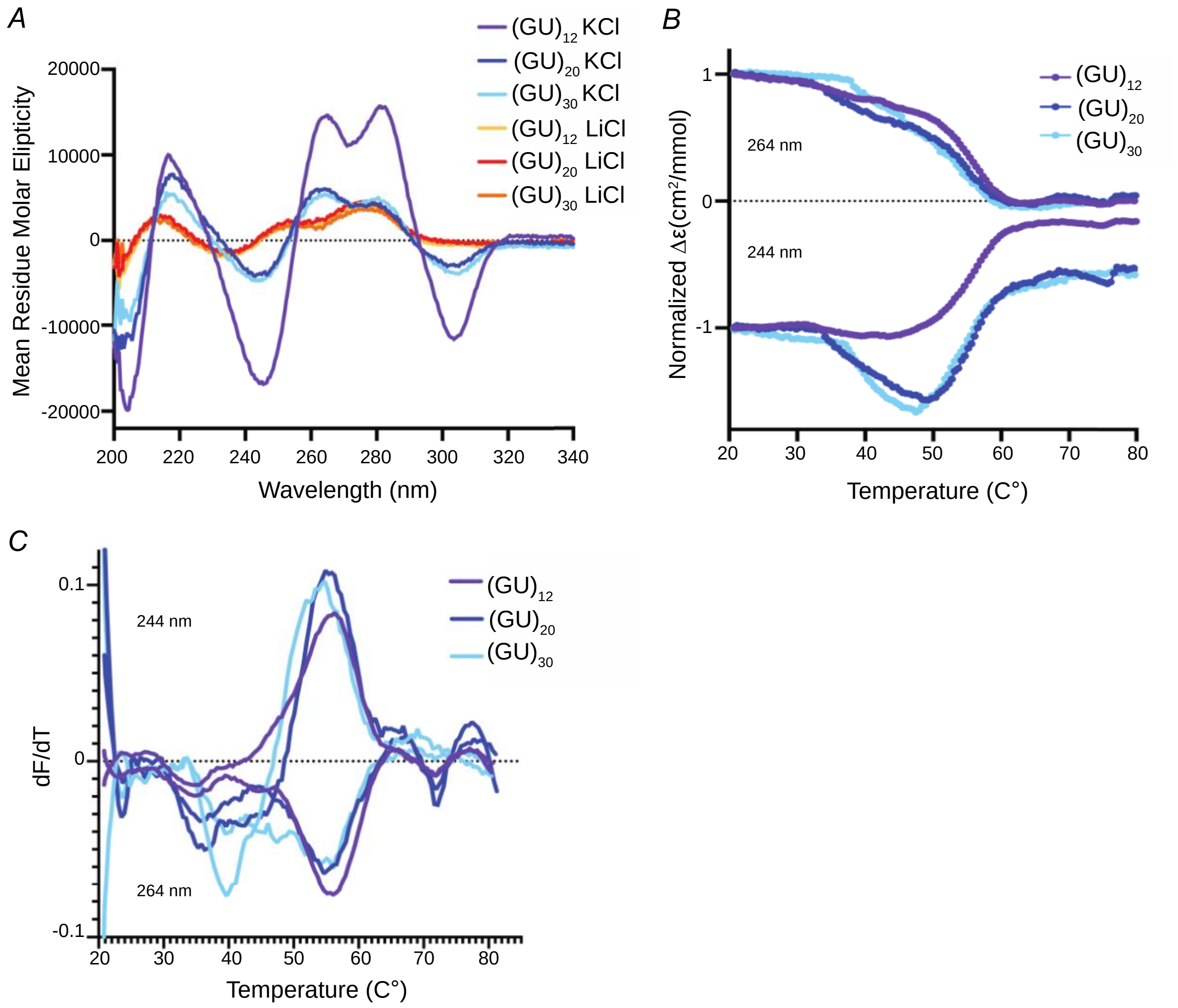

Supplemental Figure S6. pUG-fold RNAs have two-step melting curves. (*A*) CD spectra of indicated RNAs in 100 mM KCl or LiCl. (*B*) Thermal melting at 264 and 244 nm for each indicated RNA. The change in molar ellipticity (Δε) has been normalized to 1 for 264 nm curves and to -1 for 244 nm curves for each RNA to enable direct comparison. Thermal melts at 264 nm were performed three different times with similar results. Thermal melts at 244 nm were performed twice with similar results. Representative melting curves for each RNA are shown. (*C*) First derivative curves for each thermal melt in (*B*). The major melting transition for each RNA is around 55 °C.

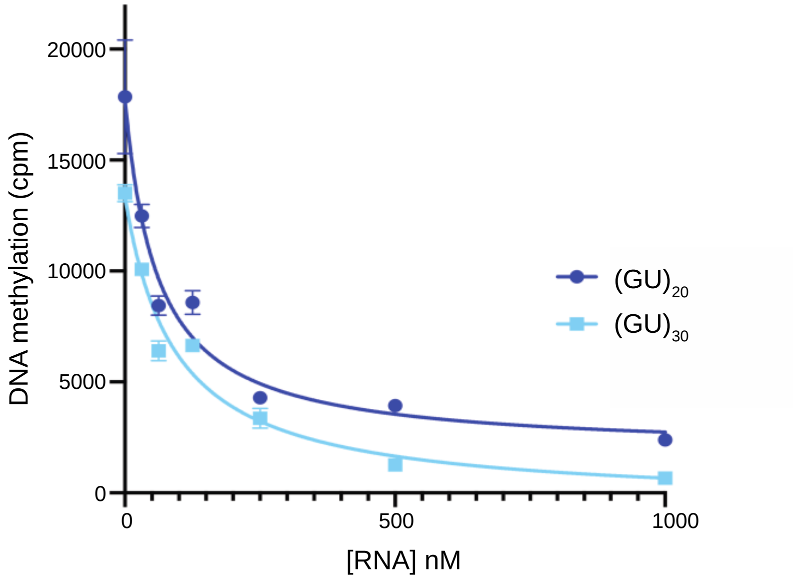

Supplemental Figure S7. DNMT1 is inhibited by two pUG-fold RNAs. DNA methylation (cpm) as a function of RNA concentration for $(GU)_{20}$ (dark blue) and $(GU)_{30}$ (light blue). Points give mean values, error bars give SD for n = 3.

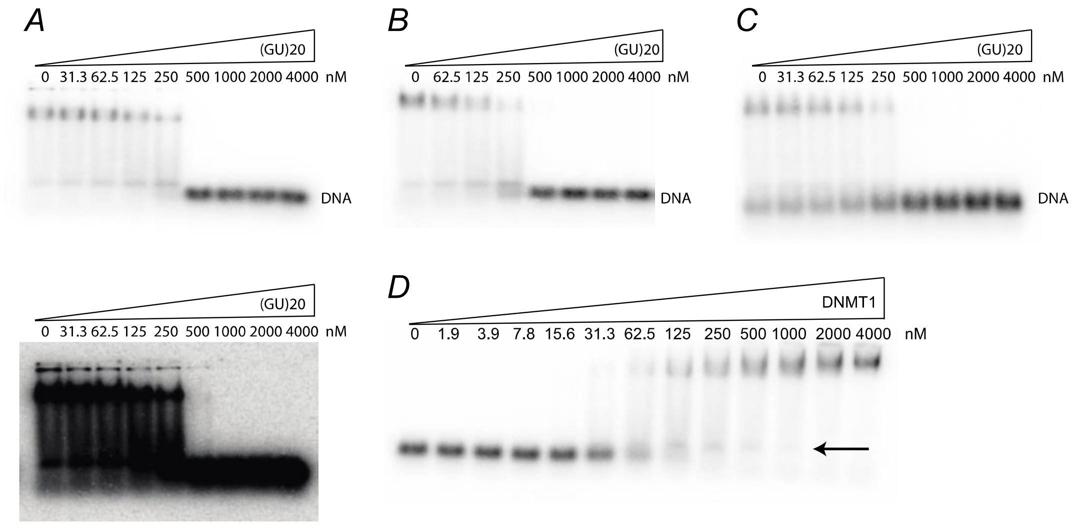

Supplemental Figure S8. $(GU)_{20}$ competes with hemimethylated DNA for DNMT1 binding. *(*A) Original competition EMSA shown in Figure 5C (top panel), with a darker contrast of the same gel shown in the bottom panel. (*B*) and (*C*) A second and third independent replicate of the experiment shown in (*A*). The gel in (*B*) is missing the sample for the 31.3 nM DNMT1 lane. The gel in (*C*) was performed with a DNMT1 aliquot that was at a lower concentration than the DNMT1 used for gels shown in (*A*) and (*B*); this is reflected by the higher fraction of unbound DNA across lanes. (*D*) EMSA of hemimethylated DNA bound to DNA to illustrate the presence of a DNA band (arrow) that runs slightly above the unbound DNA band (appears around 125 nM DNMT1) to demonstrate why the "unbound" DNA in the competition EMSA looks different in the DNA-bound vs unbound lanes (compare lanes 250 nM and 500 nM in (*A*)).

WT DNMT1 3’UTR
5’UUCUGCCCUCCCGUCACCCCUGUUUCUGGCACCAGGAAUCCCCAACAUGCACUG
AUGUUGUGUUUUUAACAUGUCAAUCUGUCCGUUCACAUGUGUGGUACAUGGUGU
UUGUGGCCUUGGCUGACAUGAAGCUGUUGUGUGAGGUUCGCUUAUCAACUAAUG
AUUUAGUGAUCAAAUUGUGCAGUACUUUGUGCAUUCUGGAUUUUAAAAGUUUUU
UAUUAUGCAUUAUAUCAAAUCUACCACUGUAUGAGUGGAAAUUAAGACUUUAUG
UAGUUUUUAUAUGUUGUAAUAUUUCUUCAAAUAAAUCUCUCCUAUAAACCA 3’

GU mutant
5’UUCCACCCUCCCACCACCCCUACUUCCAGCACCAGGAAUCCCCAACACACACCAA
CAUCACAUUUUUAACACAUCAAUCUACCCACUCACACACACAACACACAACACUU
ACGGCCUCAGCCAACACAAAGCCAUCACACAAGACUCGCUUAUCAACUAACAAUU
UAACGAUCAAAUCACACAACACUUUACGCAUUCCAGAUUUUAAAAACUUUUUAU
UACACAUUAUAUCAAAUCUACCACCAUACAAGCAGAAAUUAAGACUUUACAUAA
CUUUUAUACAUUACAAUAUUUCUUCAAAUAAAUCUCUCCUAUAAACCA 3’

GU stretch mutant
5’UUCUGCCCUCCCGUCACCCCUGUUUCUGGCACCAGGAAUCCCCAACAUGCACUG
ACACCACACUUUUAACAUGUCAAUCUGUCCGUUCACACACACAACACACAACACC
CACAGCCUUGGCUGACAUGAAGCCACCACACAAGGUUCGCUUAUCAACUAAUGAU
UUAGUGAUCAAAUUGUGCAGUACUUUGUGCAUUCUGGAUUUUAAAAGUUUUUUA
UUAUGCAUUAUAUCAAAUCUACCACUGUAUGAGUGGAAAUUAAGACUUUAUGUA
GUUUUUAUAUGUUGUAAUAUUUCUUCAAAUAAAUCUCUCCUAUAAACCA 3’

Supplemental Figure S9. DNMT1 3’UTR mutants. GU/UG dinucleotides in the WT 3’UTR are highlighted in yellow (upper panel). Mutated nucleotides in the GU mutant are indicated by lack of UG/GU highlighting (middle panel). Mutated consecutive GU/UG stretches in the GU stretch mutant are highlighted in yellow (bottom panel).

RNA/DNA	Sequence
RNA3	GGGAAUGGCAGAUGCCAACAGCCCCCCCAAACCCCUUCCAAACCUCGCACGCCCAGGAGGAGCAAGUCCGAUGGAGAGGCUAAGCGUU CAAGAGACCCUCCUGCCUCAGCCUCCCAAGUAACUGGGAUUAGAGCUGAACCUUCACCUAGCCCCAGGAUUACAAGGAAAAGCACCAG GCAAACCACCAUCACAUCUCAUUUUGCAAAG
RNA7	GGGCAGUACCUGGACGACCCUGACCUCAAAUAUGGGCAGCACCCACCAGACGCGGUGGAUGAGCCACAGAUGCUGACAAAUGAGAAGC UGUCCAUCUUUGAUGCCAACGAGUCUGGCUUUGAGAGUUAUGAGGCGCUUCCCCAGCACAAACUGACCUGCUUCAGUGUGUACUGU AAGCACGGUCACCUGUGUCCCAUCGACACCGGCCUCAUCGAGAAGAAUAUCGAACUCUUCUUUUCUGGUUCAGCAAAAC
RNA12	GGAAGUCGAUGAUAACAUCCCAGAGAUGCCGUCACCCAAAAAAAUGCACCAGGGGAAGAAGAAGAAACAGAACAAGAAUCGCAUCUCU UGGGUCGGAGAAGCCGUCAAGACUGAUGGGAAGAAGAGUUACUAUAAGAAGGUGUGCAUUGAUGCGGAAACCCUGGAAGUGGGGG ACUGUGUCUCUGUUAUUCCAGAUGAUUCCUCAAAACCGCUGUAUCUAGCAAG
RNA13	GGGACUGUGUCUCUGUUAUUCCAGAUGAUUCCUCAAAACCGCUGUAUCUAGCAAGGGGACUGUGUCUCUGUUAUUCCAGAUGAUUC CUCAAAACCGCUGUAUCUAGCAAGGGUCACGGCGCUGUGGGAGGACAGCAGCAACGGGCAGAUGUUUCACGCCCACUGGUUCUGCGC UGGGACAGACACAGUCCUCGGGGCCACGUCGGACCCUCUGGAGCUGUUCUUGGUGGAUGAAUGUGAGGACAUGCAGCUUUCAUAUA UCCACAGCAAAGUGAAAG
RNA23	GGGACCACAUCUGUAAGGACAUGAGUGCAUUGGUGGCUGCCCGCAUGCGGCACAUCCCCUUGGCCCCAGGGUCAGACUGGCGCGAUC UGCCCAACAUCGAGGUGCGGCUCUCAGACGGCACCAUGGCCAGGAAGCUGCGGUAUACCCACCAUGACAGGAAGAACGGCCGCAGCAG CUCUGGGGCCCUCCGUGGGGUCUGCUCCUG
RNA25	GGGAGUGUGCCCGCUCCCAGGGCUUCCCUGACACCUACCGGCUCUUCGGCAACAUCCUGGACAAGCACCGGCAGGUGGGCAAUGCCG UGCCACCGCCCCUGGCCAAAGCCAUUGGCUUGGAGAUCAAGCUUUGUAUGUUGGCCAAAGCCCGAGAGAGUGCCUCAGCUAAAAUAA AGGAGGAGGAAGCUGCUAAGGACUAG
$(C)_{40}$	$(C)_{40}$
$(G)_{40}$	$(G)_{40}$
$(U)_{40}$	$(U)_{40}$
$(A)_{40}$	$(A)_{40}$
$(GA)_{20}$	$(GA)_{20}$
$(GGAA)_{10}$	$(GGAA)_{10}$
$(CGG)_{12}$	$(CGG)_{12}$
$(GU)_{20}$	$(GU)_{20}$
4 nt GU loop	CUUUCCAAACCUCGCACCGUGUGGUGCGAGGUUUGGAAAG
4 nt UUCG loop	CUUUCCAAACCUCGCACCUUCGGGUGCGAGGUUUGGAAAG
8 nt GU loop	CUUCCAAACCUCGCACAAGUGUAAGUGCGAGGUUUGGAAG
R5*	CUGAGGCCUUGGCGAGGCUUCU
R4*	UCACACACACCGCAGCUCCAGA
$(GU)_{11}$	$(GU)_{11}$
$(GU)_{12}$	$(GU)_{12}$
$(GU)_{30}$	$(GU)_{30}$
$(AC)_{20}$	$(AC)_{20}$
$(AC)_{30}$	$(AC)_{30}$
3' UTR	UUCUGCCCUCCCGUCACCCCUGUUUCUGGCACCAGGAAUCCCCAACAUGCACUGAUGUUGUGUUUUUAACAUGUCAAUCUGUCCGUU CACAUGUGUGGUACAUGGUGUUUGUGGCCUUGGCUGACAUGAAGCUGUUGUGUGAGGUUCGCUUAUCAACUAAUGAUUUAGUGA UCAAAUUGUGCAGUACUUUGUGCAUUCUGGAUUUUAAAAGUUUUUUAUUAUGCAUUAUAUCAAAUCUACCACUGUAUGAGUGGAA AUUAAGACUUUAUGUAGUUUUUAUAUGUUGUAAUAUUUCUUCAAAUAAAUCUCUCCUAUAAACCA
3' UTR GU mut	UUCCACCCUCCCACCACCCCUACUUCCAGCACCAGGAAUCCCCAACACACACCAACAUCACAUUUUUAACACAUCAAUCUACCCACUCAC ACACACAACACACAACACUUACGGCCUCAGCCAACACAAAGCCAUCACACAAGACUCGCUUAUCAACUAACAAUUUAACGAUCAAAUCAC ACAACACUUUACGCAUUCCAGAUUUUAAAAACUUUUUAUUACACAUUAUAUCAAAUCUACCACCAUACAAGCAGAAAUUAAGACUUU ACAUAACUUUUAUACAUUACAAUAUUUCUUCAAAUAAAUCUCUCCUAUAAACCA
3' UTR GU stretch mut	UUCUGCCCUCCCGUCACCCCUGUUUCUGGCACCAGGAAUCCCCAACAUGCACUGACACCACACUUUUAACAUGUCAAUCUGUCCGUUC ACACACACAACACACAACACCCACAGCCUUGGCUGACAUGAAGCCACCACACAAGGUUCGCUUAUCAACUAAUGAUUUAGUGAUCAAA UUGUGCAGUACUUUGUGCAUUCUGGAUUUUAAAAGUUUUUUAUUAUGCAUUAUAUCAAAUCUACCACUGUAUGAGUGGAAAUUAA GACUUUAUGUAGUUUUUAUAUGUUGUAAUAUUUCUUCAAAUAAAUCUCUCCUAUAAACCA
hemi-methylated DNA substrate	TA(5mC)GTATC(5mC)GTATC(5mC)GGTTA(5mC)GTATC(5mC)GAATC(5mC)GTAC(5mC)GT / ACGGTACGGATTCGGATACGTAACCGGATACGGATACGTA

Supplemental Table S1. Sequences of all nucleic acid constructs used in this study.
* (Di Ruscio et al. 2013)
